# Expeditious numerical capacity assessment in precast structures via inelastic performance-based spectra

**DOI:** 10.1016/j.heliyon.2024.e39729

**Published:** 2024-10-25

**Authors:** R. Nascimbene, E. Brunesi, A. Sisti

**Affiliations:** a Scuola Universitaria Superiore IUSS Pavia, Piazza della Vittoria n.15, 27100, Pavia, Italy; b EUCENTRE, European Centre for Training and Research in Earthquake Engineering, Via Ferrata 1, 27100, Pavia, Italy; c Rose School, Scuola Universitaria Superiore IUSS Pavia, Piazza della Vittoria n.15, 27100, Pavia, Italy

**Keywords:** P-Delta effects, Second-order effects, Precast RC hinged frames, Equivalent single-degree-of-freedom system, Incremental dynamic analysis

## Abstract

The seismic design of precast structures hinges on unique characteristics intrinsic to precast technology. Emphasis is placed on lightweight structural elements for efficient on-site assembly and cost reduction. This leads to increased slenderness in beams and columns compared to traditional cast-in-situ constructions, accentuating the role of second-order effects. Dry pinned joints, favoured for connecting beams and columns, contribute to the overall efficiency in assemblage. Cast-in-situ concrete is typically reserved for connection to foundations and topping precast floor elements. Pinned joints transform the structure into an ideal isostatic system, with cantilevered columns anchored securely at the base, in designers' mind. However, this transformation reduces the energy dissipation capacity, preventing plastic hinge formation in beams and amplifying P-Delta effects in columns. The simplified approach proposed herein assesses dynamic instability in single and multi-storey precast hinged frames, providing a tool for expeditious numerical capacity assessment, useful at the initial design stage. The goal is to predict dynamic collapse and/or the attainment of specific seismic-oriented limit states based on fundamental structural parameters. Incremental nonlinear dynamic analysis, utilising far-field ground motion records, is employed to evaluate performance of a variety of precast structures modelled as equivalent single-degree-of-freedom systems. The outcomes yield inelastic spectra that provide insights into the structural capacity in terms of response modification factor and could help analysts/designers towards seismic performance-based design as well as the assessment problem. These spectra, which can themselves be taken as metrics for structural performance evaluation (in addition to as a reliable tool/means for design), are generated based on various structural parameters, including building height, column aspect ratios, and floor mass configurations, in relation to different limit states typically deemed crucial in the design of these structures for earthquake-induced actions. Regression analyses of median spectra show that polynomial expressions could fit them with good accuracy, as testified by a coefficient of determination in the 0.76–0.98 range for most of the cases.

## 1 Introduction

Precast concrete structures have gained extensive popularity in European and American building industries over the past few decades [1]. Their success can be attributed to several key factors: relatively low construction costs, rapid construction pace, and the ease of achieving large spans and heights. Industrial buildings, where construction speed is of paramount importance, and environmental sustainability is a priority, are prime examples of this trend [2,3]. Moreover, there are additional aspects of prefabrication closely tied to increased productivity and the significant roles played by construction costs and quality [4]. Consequently, the development of such technologies often mirrors the challenges encountered in the country where they are initially introduced. In regions with a high seismic hazard, the emphasis in design shifts towards seismic performance, with construction speed requirements taking a back seat. Conversely, in areas with lower seismic activity or where seismic events are less strongly felt, economic considerations drive the preference for swift assembly and the reduction of cast-in-situ elements.

Many precast structures in earthquake-prone countries were designed prior to the widespread adoption of modern seismic design concepts and current seismic design classifications [5,6]. Nevertheless, these buildings continue to operate in regions with moderate to high seismic activity. Interestingly, even precast structures designed within the last decade appear to lag behind the continuously evolving seismic design requirements for conventional cast-in-place concrete buildings. Recent earthquakes in Italy (such as L'Aquila in 2009 and Emilia-Romagna in 2012) [[7], [8], [9], [10], [11], [12], [13], [14]] and in Turkey (including Kocaeli-Duzce in 1999 and Van in 2011, as well as the Pazarcik and Elbistan districts of Kahramanmaras in 2023) ([15,16]; [61]; [[17], [18], [19], [20]]) have provided unfortunate examples and have underscored the urgent need for a comprehensive campaign to assess and retrofit this type of structures.

**Fig. 1:**
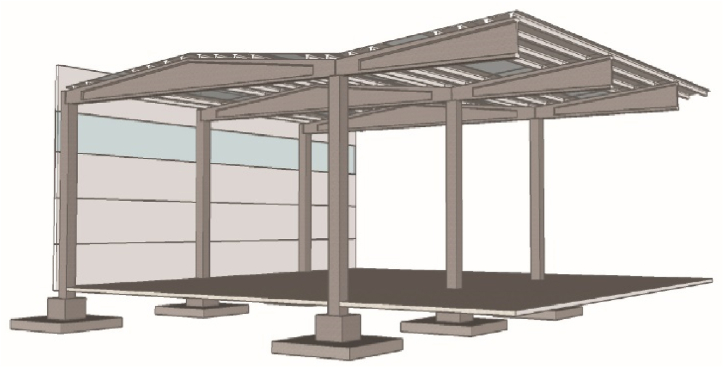
Qualitative schematic example of a typical single-storey multi-bay precast structure.

Typically (Fig. 1), these structures are characterised by single-storey, multi-bay configurations with “hinged” or “pinned” frames in the transverse direction. These frames feature slender columns anchored in individual foundations and beams with spans ranging from 14 to 20 m, often incorporating pre-stressed components. The beams are connected to the columns through semi-rigid beam-column connections. In the longitudinal direction, there is frequently no additional lateral-load resisting system. Although it is a common misconception that the columns and the roof/diaphragm form a lateral resisting frame system, the distribution of horizontal forces is determined by the relative strength of the columns, connections, and the stiffness of the roof diaphragm.

This research is primarily concerned with slender precast structures constructed using precast concrete frames featuring pinned-end or pin-ended beams. The seismic design of these structures is significantly influenced by the inherent characteristics of precast technology. Given that a pivotal concern is to provide structural elements that are as lightweight as possible to simplify on-site assembly and reduce costs, the beams and columns may become notably slimmer compared to traditional cast-in-situ concrete constructions. Consequently, second-order effects become of paramount importance in the design process. Furthermore, for the same reasons mentioned above, designers often prefer the use of dry pinned joints to connect beams and columns. Cast-in-situ concrete is typically reserved for column-to-foundation connections [21] and topping off precast floor elements [22]. Pinned joints effectively transform the frame into an ideal isostatic structure with cantilevered columns anchored at the base. This transformation leads to a substantial reduction in the structure's capacity to dissipate energy, thus preventing the formation of plastic hinges in the beams, which is a (well-accepted) stable and trustworthy dissipative mechanism. Simultaneously, this scenario amplifies the P-Delta effects in the column's response by decreasing the overall stiffness when subjected to lateral loads. Considering the factors outlined above, the seismic design of precast frames with pinned beam-column connections is significantly more reliant on second-order effects compared to traditional cast-in-situ concrete ones. Consequently, the seismic design approach for these structures differs substantially from that for cast-in-situ solutions. In precast hinged frames, there is a heightened emphasis on drift limits at serviceability limit states in contrast to ductility demand in plastic hinges. Additionally, it becomes imperative to account for dynamic instability at collapse or near collapse or collapse prevention limit states.

In this paper, a simplified approach is presented for evaluating dynamic instability in single- and multi-storey precast hinged frames. The primary objective is to provide a tool for the expeditious assessment and/or preliminary seismic design of such structures, enabling the prediction of dynamic collapse and the attainment of key limit states under seismic excitation using fundamental structural parameters. The contributions of these parameters to the overall structural behaviour are explored through nonlinear incremental dynamic analysis (IDA) using a set of 15 far-field ground motion records and case-study structures modelled as equivalent single-degree-of-freedom (ESDOF) systems. The outcome of this research yields sets of inelastic spectra that provide insights into the structural capacity in terms of response modification factor or force reduction factor defined later in the manuscript according to according Adam and Jager [23] and Ibarra and Krawinkler [24]. These spectra are generated based on various parameters, including building height, column aspect ratios, and floor mass configurations, in relation to different limit states typically considered for the seismic design of these structures.

## 2 Methodology

In this Section, key methodological aspects involved in the procedure/framework for derivation of inelastic performance-based spectra are given. Numerical modelling efforts are firstly placed in a broader context by hinting at precast technology and background information related to design codes and code-compliancy of the simple yet reliable and robust/stable mechanics-based model concept (Section 2.1). Then, items involved in the assessment of P-Delta effects regarding numerical modelling and analysis criteria are presented and described in detail (Section 2.2 and Section 2.3), so as to permit a readily interpretation of sets of obtained results.

Finally, it is worth clarifying that a decision has been made to consider ESDOF systems, explaining in Section 2.2.2 each and every step for passing from reference structure to equivalent multi-degree-of-freedom (EMDOF) systems to ESDOF ones (and vice versa), which is also a transparent means for telling the difference of precast configurations involved and/or for identifying the structure of interest, notwithstanding that all models and, hence, structural configurations refer to the case of pin-ended beams, to which dry dowel connections well adapt.

### 2.1 Past earthquakes and current design approach in precast technology

On May 20, 2012, a magnitude 5.9 earthquake struck the Emilia-Romagna region in Northern Italy. This event resulted in 7 fatalities, 50 injuries, and approximately 5000 people left homeless. Following a series of minor aftershocks, another earthquake with a magnitude of 5.8 ($M_{w}$) hit the same region on May 29, with its epicenter located just 20 km away from the initial event.

These two seismic events inflicted extensive damage on existing structures, particularly precast concrete industrial buildings and historic masonry structures. Focussing on the former category, the observed significant damage can be attributed to a couple of main factors [7,10]: the high number of precast structures because the region impacted by the earthquakes had a substantial concentration of precast structures, and their elevated vulnerability.

The first point is related to the fact that the earthquake sequence affected a vital industrial district in Italy, where numerous industrial buildings were constructed. However, the majority of these industrial buildings were built without seismic design provisions because mandatory seismic design requirements in the affected area were only introduced in 2005, which significantly contributed to the vulnerability of these structures during the abovementioned seismic events. Thus, for this reason, most of the observed damage was related to the delay in the adoption and implementation of adequate seismic provisions, as well as to the mistrust towards new seismically efficient, but more complex, solutions [7]. Interested readers are kindly referred to Belleri et al. [7] and Magliulo et al. [10], amongst others, for what concerns the reason behind the lack of seismic design for this kind of structures, and – most regrettably – the severe consequences and implications this had in this past earthquake sequence.

Prior to the implementation of seismic design codes, the typical practice in the precast building industry involved the use of slender beam and column elements with friction-based connections. The following main mechanisms can be recognised [25,26]: loss of support between roof elements and main beams; loss of support between beams and columns; loss of support of beam by lateral sliding for lack of restraint; failure of cladding panel connections. Contributions to these mechanisms were also provided by the following factors: interaction with non-structural masonry elements; inner column plastic hinging at the base; lack of rigid floor diaphragms; base socket foundation rigid rotation (foundation failure). It is worth noting that the majority of collapses have been due to high flexibility of the structures and lack of adequate connections among the elements. The significant displacement of the column tops, which was accentuated by second-order effects due to the slenderness of the structural elements, played a pivotal role in causing the loss of support for the main beams. Such a large displacement also resulted in a substantial interaction with non-structural components, particularly in cases where these elements were irregularly positioned or inadequately designed. These interactions had a detrimental impact on the overall structural response, exacerbating the vulnerability of the precast structures during these seismic events. Additionally, the significant deformations experienced during earthquakes also contributed to the failure of connections between cladding panels and their supporting elements, such as beams and columns. The observations above underscore the critical importance of assessing flexibility and P-Delta effects when dealing with the seismic design of precast hinged structures.

The current approach to the design of hinged frames takes into account the insights derived from the research mentioned earlier, as well as on-site experience and field observation of failure. This approach aligns with the principles of force-based design (FBD) [27], which remains the most commonly used approach in building codes worldwide. Seismic-resistant structures, in a FBD framework, are designed to satisfy two primary families of limit states [60]: ultimate limit states (ULSs) and damage limitation states (DLSs).

In the following Sections, ULS design is conducted through the use of both the inelastic spectra approach and multimodal linear analysis (MMA). This method relies upon behaviour factors that capture the ductility and dissipative capacity of each category of structures. It becomes necessary, at ULSs, to consider P-Delta effects when their influence becomes significant. Eurocode 8 [60] provides a simple criterion for considering P-Delta effects, which is based on the stability index θ:

(1) $$\theta =\frac{P_{TOT}\cdot d_{r}}{V_{TOT}\cdot h}$$

where: $P_{TOT}$ is the total gravity load at (and above) the storey level considered, in seismic combination; $d_{r}$ is the design inter-storey drift at the considered ULS; $V_{TOT}$ is the total seismic storey shear; and h is the inter-storey height.

Evaluations of DLSs primarily concentrate on the examination of damage to non-structural components and/or secondary elements within the building such as cladding panels and/or infills. To ensure the integrity of these components, limitations are typically imposed on inter-storey drifts, based on the fragility of partitions and other non-structural elements [[28], [29], [30]]. These limitations are evaluated for a level of seismic action lower than that used for ULS design, as the aim is to prevent damage that could compromise the functionality and usability of the building without necessarily causing structural failure. Eurocode 8 [60] provides specific limitations for DLS as follows.

• for buildings having non-structural elements of brittle materials attached to the structures:

(2) $$d_{r}<0.005\cdot h$$

• for buildings having ductile non-structural elements:

(3) $$d_{r}<0.0075\cdot h$$

• for buildings having non-structural elements fixed in a way that they do not interfere with structural deformations or without non-structural elements:

(4) $$d_{r}<0.01\cdot h$$

Similar values are provided in many other building codes, as many embrace this same philosophy for the design. For instance, the Italian Building Code, NTC18 [31], provides the exact same values of inter-storey drifts embracing the exact same philosophy. To name another one, ACI 318-19 [32] provides inter-storey drift limits of 0.005ℎ and 0.01ℎ for the case of non-prestressed slabs and unbonded post-tensioned slabs, respectively, when referring to slab-column connections – see Clause 18.14.5.2 of that document [32]. Other criteria for controlling drifts at the ultimate limit state are also provided therein, expectedly implying much larger drifts or design storey drift ratios, to comply with ACI 318 terminology. Also, the same code in a former version/release suggests the larger of 0.005ℎ and an expression function of the factored shear force – see Clause 21.11.5(b) in ACI 318-05 [33].

Furthermore, FEMA356 [34] provides component-specific – e.g. infill-specific – drift limits for different limit states to be considered in the seismic design or assessment or retrofit problem. Other design codes such as ASCE 7 [35] and NZS 1170 [36] specify typical storey drift ratio limits of 2–2.5 % for building structures under design-level seismic actions. Furthermore, deformation limits on component level (i.e. rotation limit), section level (i.e. curvature limit), or material level (i.e. strain limit) are also provided by different design documents. As previously alluded to, ACI 318-19 [32] is one of those as it gives component-level deformation limits for concrete members (e.g. walls); limits at the component and section levels for different components are also provided in and the LATBSDC [37]. The New Zealand NZS 3101:2006 [38] is another example, as it provides section-level deformation limits in the form of normalised curvature ductility, with the latter being set to correspond to lower-bound limits for the ultimate deformation capacity based on a 20 % drop in lateral resistance of concrete components.

Coming back to the European/Italian context, the limit given in (4) usually applies to cladding panels in precast concrete structures, while limits in (2) and (3) typically refer to clay masonry claddings or wall plugs. For the purpose of this research, a key aspect to emphasise in the seismic design of precast hinged frames, especially when compared to other types of concrete structures, is the substantial influence of provisions related to P-Delta effects on the design process. This influence remains significant even when the seismic action is moderate, and the structures are low-rise ones. In many cases, P-Delta effects impose the most stringent limitation on the geometry of structural elements, which comes as a direct result of the inherent flexibility of the precast hinged frame system.

### 2.2 Assessment of P-Delta effects in precast hinged frames

As highlighted in the preceding Section, structural design is predominantly influenced by second-order effects, often requiring iterations to achieve a feasible solution. This involves initially verifying DLS requirements, assessing capacity at the ULS, and subsequently ensuring control over the stability index value.

**Fig. 2:**
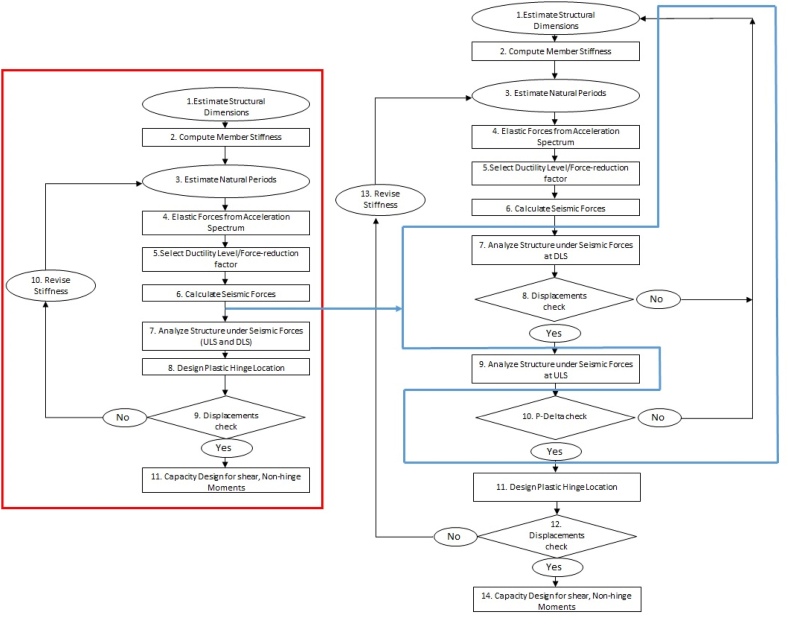
Typical reinforced concrete FBD procedure (left) and modified precast hinged frame design procedure driven by displacements rather than forces (right).

This procedure is complex and time-consuming, requiring, typically, several analysis also for relatively simple structures. Hence, it can be useful to elaborate an analytical approach to checking relevant limit states in preliminary stages of the design process, starting from simple geometric and mechanical parameters, which is one of the main aims of this research. To clarify advancements compared to the previously outlined state-of-the-art methodologies, a comprehensive flowchart is presented in Fig. 2. The left side of the diagram, highlighted in red, delineates the FBD process. On the right side, a newly introduced chart depicting key steps for precast hinged frame systems is outlined and distinguished by a distinctive blue color. In particular, Step 9 involves conducting a displacement check, which is crucial for correcting the approximations made when estimating the natural periods of the structure at the outset of the procedure. However, in current practice, this step is often skipped due to the time-consuming nature of the process. Instead, designers typically rely upon code-prescribed safety factors and adopt a generally conservative performance-based design approach for the structure in Step 10 to account for uncertainties of some level and sort. In the same Fig. 2 (but highlighted in blue color on the right), the corresponding chart for the studied case is presented. Here, it is important to note that an iterative process is often necessary, especially when considering both DLS attainment and the geometric requirements related to P-Delta effects.

In the force-based approach for seismic design, depicted on the left side of Fig. 2, the primary emphasis is on assessing and distributing forces within the structure. The design process involves calculating the forces induced by seismic actions and ensuring that these forces do not surpass structural capacity. However, when transitioning to a displacement-based approach on the right blue part of Fig. 2 (or to be clearer a force approach based on displacement checks, which become the main design driver), the focus shifts towards evaluating and controlling the overall displacements expected to be experienced by the structure during a seismic event. One key aspect of this approach is the consideration of P-Delta effects. These effects arise from the interaction between lateral loads and deformations, leading to additional moments that the structure need to balance. In the iterative check on displacement, particular attention is given to addressing the impact of P-Delta effects, ensuring that the structural response adequately accounts for the geometric nonlinearity induced by them. Now, when it comes to precast structures, the scenario becomes more intricate, primarily due to the presence of hinged supports. Hinged supports imply a greater degree of flexibility in the structure's response to seismic demand. In precast hinged frames, the joints allow for extra rotation, contributing to a more flexible response. This increased flexibility is a distinctive feature that sets precast hinged structures apart from traditional monolithic reinforced concrete constructions. The iterative checks on displacement (highlighted in blue color) become particularly crucial in precast structures with hinged supports. The design process needs to account for the dynamic behaviour of these joints and the potential redistribution of forces and moments as the structure undergoes deformation. Precast elements, being manufactured separately and assembled on-site, introduce additional considerations in terms of joint detailing and compatibility, further influencing the displacement-based design approach.

In summary, the transition from a force-based to an approach based on displacement checks involves a shift in focussing towards the control of displacements, with a heightened awareness of P-Delta effects. When dealing with precast structures featuring hinged supports, this shift becomes more intricate due to the inherent flexibility introduced by the joints, necessitating a nuanced consideration of the dynamic behaviour and compatibility of the precast elements in the seismic design process.

Along this line, an interesting approach to the assessment of P-Delta effects is the development of collapse capacity spectra (CCS) [23,[39], [40], [41]]. This procedure was elaborated primarily for evaluating the susceptibility to dynamic instability of traditional/ordinary reinforced concrete and steel tall structures under earthquake events, by means of diagrams and/or charts providing the collapse capacities, in terms of response modification factor defined in accordance with Adam and Jager [23] and Ibarra and Krawinkler [24] versus the natural elastic period of the studied nonlinear single-degree-of-freedom (SDOF) system, given the stability ratio θ, the post-yielding hardening ratio α, and hysteretic cycle of the elasto-plastic hinge at the base.

Despite its widespread success in predicting dynamic instability, employing an ESDOF approximation in structural modelling presents potential shortcomings [39], specifically associated with multi-degree-of-freedom (MDOF) effects [42]. Addressing these concerns is a pivotal advancement in this research, outlined as follows.

1. the accuracy of predicting structural behaviour using ESDOF systems diminishes with an increasing number of storeys, primarily due to higher modes effects. This impact is notably observed in drifts and sectional forces, resulting in reduced global deformations;
2. dynamic forces induced by higher modes can initiate inelastic mechanisms that deviate from the assumptions made in ESDOF. It is noteworthy that this issue is more pertinent to existing buildings, as performance-based designed structures generally permit only a single plastic mechanism through precise strength allocation.

The first concern is of paramount importance for design methods. Evaluating these effects allows for a comparison with the degree of approximation in the assumed model, encompassing second-order effects, their correlations, ductility demand, and expected inelastic mechanisms.

Based on these considerations, particularly those arising from MDOF-related effects, a procedure akin to the one employed in CCS computations is formulated and tested. The objective is to extract inelastic spectra for various limit states crucial in the design of precast structures. As such, relevant limit states are defined, a method for determining ESDOF characteristics is applied and discussed, and the framework for IDA is presented. This approach enables the utilisation of inelastic spectra, establishing correlations between energy components and the corresponding maximum displacement response parameters of the structure (Proietti et al. [43]. As a result, it becomes possible to directly predict and control the maximum seismic energy demand that a structure needs to dissipate, employing a simplified approach, while discerning the different components of the energy balance, including the hysteretic one.

#### 2.2.1 Definition of limit states

Moving on with the procedure, originally developed in Adam and Jager [23], specific spectra, which are the focal point of the work presented, have to be derived. These spectra are categorised into two main groups: limit state spectra (LSS) and drift limit spectra (DLS).

(a) **Limit state spectra (LSS).** These spectra can be uniquely defined to deal with different limit states, and they concern the dynamic instability collapse (CC – LS3) and the attainment of material strain limit at damage control limit state (DL – LS2) and at serviceability limit state (SL – LS1). About these latter limit states, the following strain limits are considered, according to Priestley et al. [44]:
  • Damage control limit state (DL – LS2): limitations on both concrete compressive strain and steel tensile strain are imposed. The former strongly depends on the confinement provided by transverse reinforcement, so some assumptions will be needed in order to take into account such a condition in the model (e.g. the attainment of concrete compressive stress peak, conservatively, or a strain twice larger to more properly be in the softening regime of concrete material response). On the other hand, steel tensile strain at damage limit state can be assumed with good accuracy equal to $0.6\varepsilon_{su}$ [45], with $\varepsilon_{su}$ defined as the strain at maximum stress from monotonic testing endeavours (assumed here equal to 10 %);
  • Serviceability limit state (SL – LS1): concrete compressive strain limit can be assumed conservatively equal to 0.4 % to avoid spalling, while crack width can be limited to about 1 mm in members with axial compression by imposing a steel tensile strain limit of 1.5 %. These two values are assumed in determining LSS.
(b) **Drift limit spectra (DLS).** These spectra primarily address drift limits, which can regard both serviceability related to non-structural damage [46,47] and damage limit states concerning damage in the connections [[48], [49], [50]]. Therefore, although it is acknowledged that categorising these spectra a priori can be challenging due to the wide range of potential drift limit scenarios, using spectra for drift limits of 0.5 % (DR05), 1.0 % (DR10), and 2.0 % (DR20) is proposed herein to account for typical drift limits associated with the seismic behaviour of precast structural systems (especially framed ones featuring pin/dowel beam-column connections – see e.g. Ref. [51]). This approach also allows for flexibility in addressing a range of drift-related considerations in precast hinged frame design [51].

#### 2.2.2 ESDOF model

Taking into consideration the mentioned characteristics of hinged frames, it seems beneficial to incorporate the proposed approach into a DDBD framework [44]. This method has evolved over the last few decades from the most common FBD approach, aiming to address some limitations inherent in traditional seismic design methodologies. In seismic design, the early-stage control of structural displacements is achieved through DDBD, which primarily enforces limit state requirements via pre-defined deformation limits. It involves the use of equivalent substitute structures following the SDOF scheme and the overdamped spectrum method [52,53]. Subsequently, element forces are computed, and performance-based design is undertaken. The critical step in transitioning from the actual structure to ESDOFs lies in selecting an appropriate displacement profile.

**Fig. 3:**
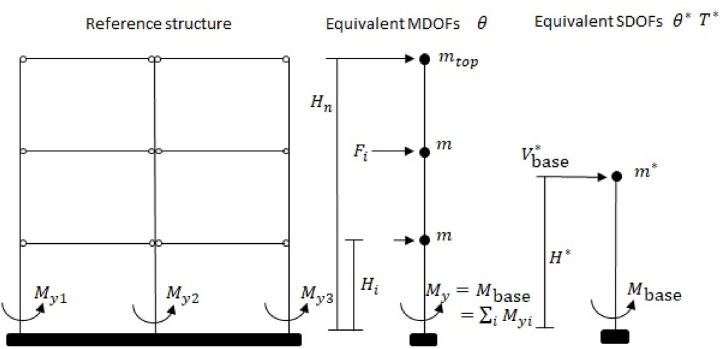
Reference frame, EMDOF system and ESDOF system.

For instance, a generic multi-storey hinged frame with columns fixed at the base is considered, as in Fig. 3. It is assumed that all columns have the same cross-section, which remains constant along the height of the frame. The inter-storey height is also assumed to be constant for all floors. However, the ratio of roof mass $m_{top}$ to floor mass m, which is assumed to be constant for all other levels, is varied within feasible limits (ranging from 100 % to 60 %). Before generating an ESDOF system, an intermediate step is necessary. Starting from the reference structure described above, an EMDOF system is derived.

One of the fundamental assumptions in DDBD for concrete structures is that the yield curvature of a cross-section depends solely on yield strain and depth of the cross-section. In this way, the assumption of having columns with same cross-section allows treating the lateral-loaded structure as an equivalent column with floor masses m lumped at each level and same total height $H_{n}$, featured by same yielding curvature $\Phi_{y}$ at the plastic hinge located at the base of the column. The yielding moment $M_{y}$ is equal to the sum of the yielding moments of the reference structure's columns at their base ($M_{yi}$). The post-yielding behaviour of plastic hinges is assumed perfectly plastic, implying no hardening is considered. Once this intermediate step is completed, the ESDOF system can be determined by applying the basic concept of substitute structure.

The structure can be simplified as a cantilevered one fixed at the base, featuring a linear curvature distribution at yielding. Although this assumption may not be entirely rigorous, especially in the case of multi-storey buildings, nonlinear analyses have demonstrated that the results are sufficiently accurate for design purposes. The displacement profile at yielding, denoted as $\Delta_{yi}$, is as follows [44]:

(5) $$\Delta_{yi}=\frac{\alpha }{2}\frac{\varepsilon_{y}H_{i}^{2}}{d}\left(1-\frac{H_{i}}{3H_{n}}\right)$$

provided the yield curvature $\Phi_{y}=\alpha \varepsilon_{y}/d$ where $\varepsilon_{y}$ is the steel reinforcement yield strain and d is the cross section depth; $H_{n}$ is the total height of the EMDOF model; α is a constant depending on the cross-section type.

**Fig. 4:**
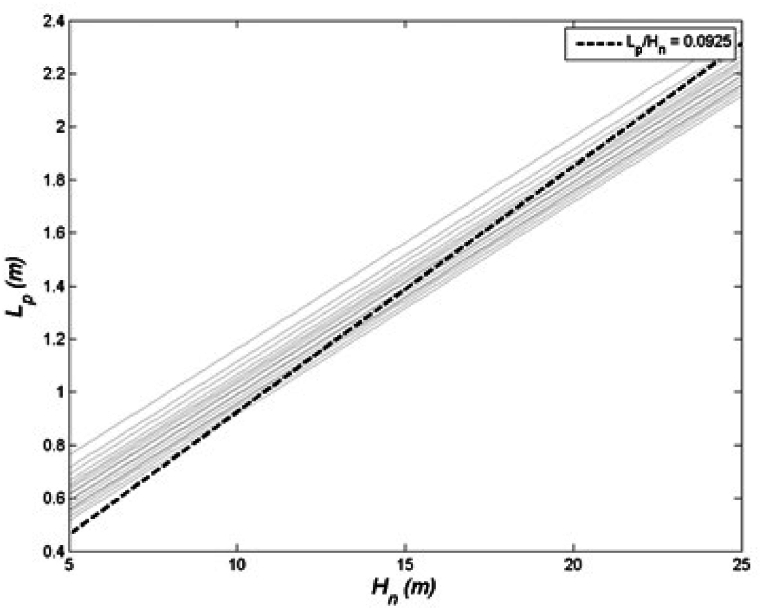
Determination of plastic hinge length coefficient β.

Plastic hinging during a severe earthquake is expected to occur at the base of the structure. This type of mechanism is characterised by a rigid rotation around the hinge located at the base of the structure, when neglecting column flexibility. The specific plastic displacement profile that emerges will depend on which damage limit state condition is reached, whether it is the drift limit or the strain limit [44]:

(6) $$\left\{ \begin{array}{c} \Delta_{pi}=\left(\Phi_{ls}-\Phi_{y}\right)L_{p}H_{i}\;\left(\text{strain}\;\text{limit}\right)\\ \Delta_{pi}=\left(\theta_{c}-\frac{\alpha }{2}\frac{\varepsilon_{y}H_{n}}{d}\right)H_{i}\;\left(\text{drift}\;\text{limit}\right) \end{array}\right.$$

where $L_{p}$ represents the plastic hinge length, which is assumed to be calculated according to Paulay and Priestley [27] as a function of steel rebar diameter $d_{b}$ and effective yield strength, the latter being taken as 1.1 times the nominal yield strength $f_{y}$ to take into account post-yielding hardening in strain penetration contribution. The first Equation (6) corresponds to strain limit attainment, while the second one corresponds to drift limit $\theta_{c}$; the value of β is determined through a process of linear regression, where pairs of $L_{p}$ and $H_{n}$ are computed within a practical range of values, and the relationship between them is analysed to find the appropriate β coefficient. More in detail, β is obtained by applying ordinary least squares (OLS) method to a sample pairs of ($H_{n}$, $L_{p}$), which are obtained by varying the values of $H_{n}$, $d_{b}$, $f_{y}$ within their practical range of interest. Assuming $H_{n}$ falls between 5m and 25m, $d_{b}$ ranges from 16 mm to 30 mm, and $f_{y}$ is within the range of 300 MPa–500 MPa, this leads to a β value of 0.0925. The results of the regression are presented in Fig. 4. The strain limit curvature in Equation (6), in DDBD of walls, can be accurately approximated as $\Phi_{ls}=\frac{k}{d}$. According to Priestley et al. [44], the parameter k holds significance, and it should be constrained to be less than 0.08. Its importance lies in its association with the ratio of ultimate tensile strength to yield strength of the flexural reinforcement. A higher value of this ratio leads to plastic deformations extending beyond the critical section, as the reinforcement at the critical section undergoes strain-hardening, consequently increasing the plastic hinge length. Conversely, if the ratio of ultimate-to-yield strength in reinforcing steel is low, plasticity tends to concentrate near the critical section, resulting in a shorter plastic hinge length. This approximation is based on observations of the curvature trend with different aspect ratios $A_{r}=H_{n}/d$ of the wall, axial forces, and reinforcement ratios, as clearly discussed by Priestley et al. [44].

Under the aforementioned assumptions, for walls, k can be considered as 0.0175 at SL – LS1 and 0.072 at DL – LS2. These values have been derived assuming that curvature limits are primarily governed by the maximum strain in the steel. However, it is important to note that these values need to be adjusted to account for precast cantilevered columns. Upon examining the diagrams and considerations given by Ref. [44], it becomes evident that dimensionless limit curvatures tend to be smaller than the assumed design limits for the highest reinforcement ratios and axial load values. Such scenarios are less common in walls but more frequent in columns. Therefore, for precast cantilevered columns, reduced values are assumed: at damage limit state DL – LS2 k is taken as 0.060, whereas at serviceability limit state SL – LS1 k is assumed to be 0.013. The idea, in this case, is simple and consists in taking, conservatively, the lower bound value of dimensionless curvature suggested by Priestley et al. [44], rather than the mean value suggested by the same scholars. For LS1 and LS2, the assumed lower bound consists in a roughly 15 % and 25 % reduction of mean dimensionless curvature.

It would be helpful to express the first Equation (6) in terms of equivalent drift by equating the second expression of Equation (6):

(7) $$\theta_{c\;\lim}=\left(k-2\varepsilon_{y}\right)\beta A_{r}+\varepsilon_{y}A_{r}$$

where $A_{r}=H_{n}/d$ represents the aspect ratio of the columns. A single relationship between the displacement profile and the drift limit can thus be established. The displacement profile for a given drift limit is determined by the combination of yielding and plastic displacement profiles using Equations (5), (6):

(8) $$\Delta_{i}=\frac{\alpha }{2}\frac{\varepsilon_{y}H_{i}^{2}}{d}\left(1-\frac{H_{i}}{3H_{n}}\right)+\left(\theta_{c}-\frac{\alpha }{2}\frac{\varepsilon_{y}H_{n}}{d}\right)H_{i}$$

Rearranging Equation (8), the displacement profile can be expressed in a dimensionless form as follows:

(9) $$\frac{\Delta_{i}}{H_{n}}=\frac{\alpha }{2}\varepsilon_{y}A_{r}{\left(\frac{H_{i}}{H_{n}}\right)}^{2}\left(1-\frac{H_{i}}{{3H}_{n}}\right)+\left(\theta_{c}-\frac{\alpha }{2}\varepsilon_{y}A_{r}\right)\frac{H_{i}}{H_{n}}$$

It is important to emphasise that the displacement shape is solely dependent on the yield strain of the steel reinforcement, the aspect ratio of the columns, and the drift limit, or equivalently, the ductility of the system.

The structural stability index (Equation (1)) is then determined based on the formulation proposed by Bolognini et al. [54], but it takes into account both displacement and shear force profiles at yielding:

(10) $$\theta =\frac{\sum_{i}P_{i}\cdot \Delta_{yi}}{\sum_{i}V_{yi}\cdot H_{i}}$$

where: $P_{i}$ represents the vertical gravity load at the i-th floor; $\Delta_{yi}$ is the displacement at the i-th floor when yielding occurs; $V_{yi}$ is the horizontal force at the i-th floor that corresponds to yielding at the base of the columns; $H_{i}$ stands for the height of the i-th floor.

The parameters of the ESDOF system/model consist of effective mass ($m^{*}$), effective height ($H^{*}$), effective base shear at yielding ($V_{\text{base}}^{*}$), effective elastic period ($T^{*}$), and effective elastic stability index ($\theta^{*}$), as illustrated in Fig. 3. The expressions for the last two parameters are as follows:

(11) $$T^{*}=2\pi \sqrt{\frac{m^{*}}{k^{*}}}$$

(12) $$\theta^{*}=\frac{m^{*}g\;\Delta_{y}^{*}}{V_{\text{base}}^{*}\;H^{*}\;}$$

where, it is needless to note that, g is the gravitational acceleration. Additionally, the remaining ones can be calculated using the following equations:

(13) $$H^{*}=\frac{\sum_{i}m_{i}\cdot H_{i}\cdot \Delta_{i}}{\sum_{i}m_{i}\cdot \Delta_{i}}$$

(14) $$m^{*}=\frac{\sum_{i}m_{i}\cdot \Delta_{i}}{\Delta^{*}}$$

(15) $$V_{\text{base}}^{*}=\frac{M_{\text{base}}}{H^{*}}$$

Equation (15) relies on the assumption that the base moment of the ESDOF system is equivalent to that of the EMDOF one. This assumption remains valid if the following force distribution for the EMDOF model is considered:

(16) $$F_{i}=V_{\text{base}}^{*}=\frac{m_{i}\cdot \Delta_{i}}{\sum_{i}m_{i}\cdot \Delta_{i}}$$

By using Equations (13), (15), (16), the moment at the base can be derived as follows:

(17) $$M_{\text{base}}=\sum_{i}F_{i}\cdot H_{i}=\sum_{i}V_{\text{base}}^{*}\frac{m_{i}\cdot H_{i}\cdot \Delta_{i}}{\sum_{i}m_{i}\cdot \Delta_{i}}=V_{\text{base}}^{*}H^{*}$$

Equation (17) illustrates the equality between the base moment of ESDOF and EMDOF systems, or, in other words, between the total base shear of the two systems. Taking into account Equation (9) and assuming a perfectly plastic hinge at the base, modifications to Equations (11), (12), (13), (14) can be made to derive valuable expressions. The subsequent relationships can thus be established:

(18) $$\frac{H^{*}}{H_{n}}=\frac{\sum_{i}\alpha_{i}\delta_{i}\frac{H_{i}}{H_{n}}}{\sum_{i}\alpha_{i}\delta_{i}}=A$$

(19) $$\frac{\Delta^{*}}{H_{n}}=\frac{\sum_{i}m_{i}\delta_{i}^{2}}{\sum_{i}m_{i}\delta_{i}}=B$$

(20) $$m^{*}=m_{\text{tot}}\frac{\sum_{i}\alpha_{i}\delta_{i}}{\frac{\Delta^{*}}{H_{n}}}=C\;{\;\cdot m}_{\text{tot}}$$

(21) $$T^{*}=2\pi \sqrt{\frac{m^{*}}{M_{\text{base}}}\cdot \frac{H^{*}}{H_{n}}\cdot \frac{\Delta_{y}^{*}}{H_{n}}}=2\pi \sqrt{\frac{m^{*}}{M_{\text{base}}}\cdot A\cdot D}$$

(22) $$\theta^{*}=\frac{m_{\text{tot}}\cdot g\cdot C\cdot D}{M_{\text{base}}}H_{n}=\frac{m_{\text{tot}}\cdot g\cdot F}{M_{\text{base}}}H_{n}$$

where:

(23) $$\alpha_{i}=\frac{m_{i}}{\sum_{i}m_{i}}=\frac{m_{i}}{m_{\text{tot}}}\;\delta_{i}=\frac{\Delta_{i}}{\Delta_{\text{roof}}}$$

with $\Delta_{\text{roof}}$ being the roof displacement and $m_{\text{tot}}$ being the total mass.

Using Equations (17), (23), the structural stability index provided in Equation (10) can be transformed into what follows:

(24) $$\theta =\frac{m_{\text{tot}}\cdot H_{n}\cdot g}{M_{\text{base}}}\sum_{i}\alpha_{i}\frac{\Delta_{yi}}{H_{n}}=\frac{m_{\text{tot}}\cdot H_{n}\cdot g}{M_{\text{base}}}E$$

Then, the following relationship applies:

(25) $$\theta =\frac{E}{F}\theta^{*}$$

**Fig. 5:**
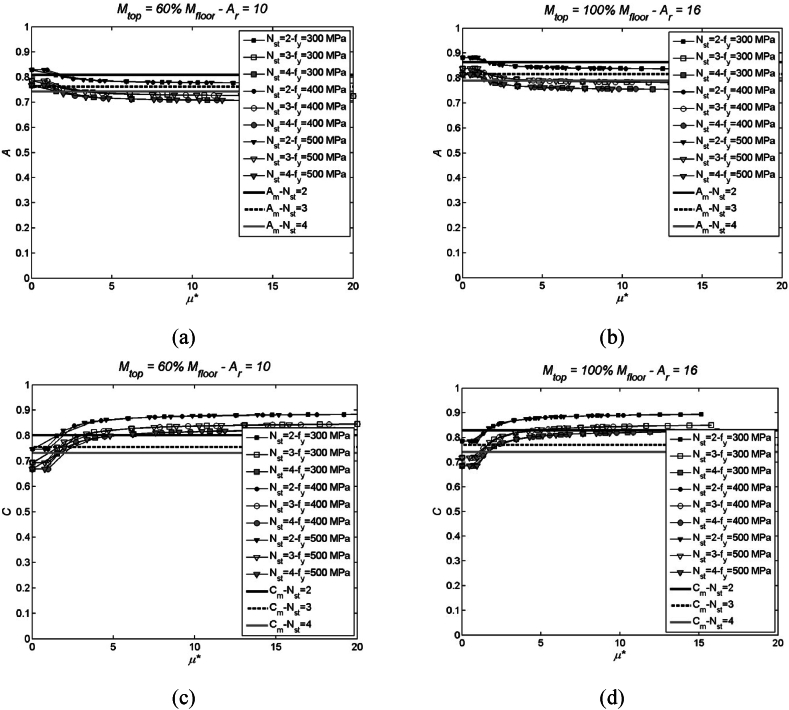
Parameters A (top) and C (bottom), and corresponding mean values, in case of $m_{top}=$ 100 % and 60 % of $m_{i}$, $A_{r}=$ 10 and 16, $f_{y}=$ 300, 400 and 500 MPa, $N_{st}$ (number of storeys) equal to 2, 3 and 4. More specifically, (a) parameter A for $m_{top}=$ 60 % of $m_{i}$ and $A_{r}=$ 10; (b) parameter A for $m_{top}=$ 100 % of $m_{i}$ and $A_{r}=$ 16; (c) parameter C for $m_{top}=$ 60 % of $m_{i}$ and $A_{r}=$ 10; (d) parameter C for $m_{top}=$ 100 % of $m_{i}$ and $A_{r}=$ 16.

**Fig. 6:**
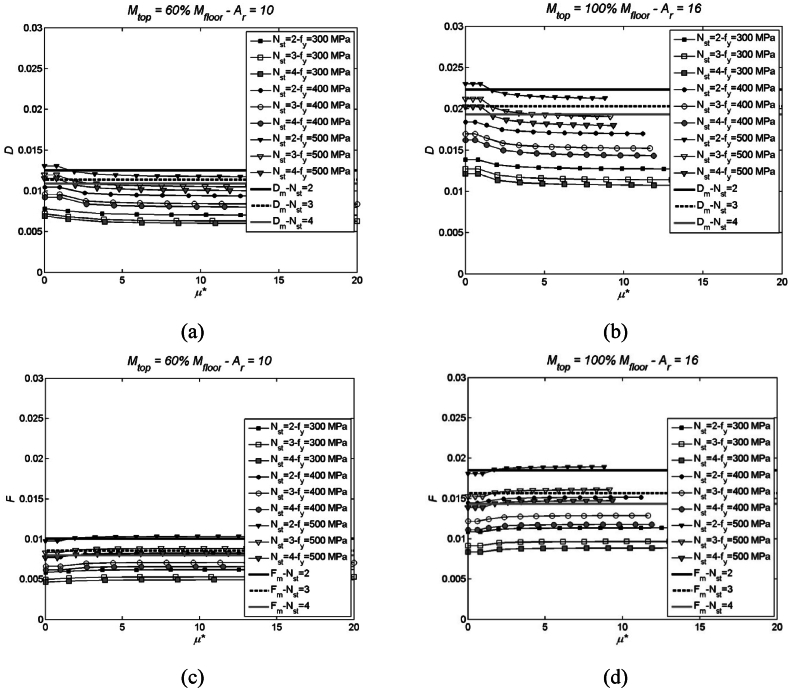
Parameters D (top) and F (bottom), and corresponding mean values, in case of $m_{top}=$ 100 % and 60 % of $m_{i}$, $A_{r}=$ 10 and 16, $f_{y}=$ 300, 400 and 500 MPa, $N_{st}$ (number of storeys) equal to 2, 3 and 4. More specifically, (a) parameter D for $m_{top}=$ 60 % of $m_{i}$ and $A_{r}=$ 10; (b) parameter D for $m_{top}=$ 100 % of $m_{i}$ and $A_{r}=$ 16; (c) parameter F for $m_{top}=$ 60 % of $m_{i}$ and $A_{r}=$ 10; (d) parameter F for $m_{top}=$ 100 % of $m_{i}$ and $A_{r}=$ 16.

The auxiliary parameters A, C, D, E, and F are not affected by the height of the structure and serve to derive the ESDOF model from fundamental structural parameters common to both reference structure and EMDOF system. However, it is worth noting that only parameters D and E remain constant regardless of the ductility of the system (as per Equations (21), (24)), whereas the other parameters vary with displacement. To facilitate the passing from EMDOF model to ESDOF one, their average values are assumed over the range of permissible ductility, typically spanning from 0 to the ductility at the strain limit/drift considered. This approach is equivalent to selecting a single displacement profile when passing from EMDOF to ESDOF system, thereby simplifying the analysis. In what follows, diagrams illustrating the variability of parameters are presented, and mean values of these parameters are indicated (see Fig. 5, Fig. 6). These parameters are calculated for a wide range of ductility levels and for various number of storeys and mass configurations. Additionally, the study explores variations in material properties. For the sake of brevity, Fig. 5, Fig. 6 only show the main diagrams related to extreme values, particularly $m_{top}=$ 100 % and 60 % of $m_{i}$, $A_{r}=$ 10 and 16, $N_{st}$ (number of storeys) equal to 2, 3 and 4; $f_{y}=$ 300, 400 and 500 MPa.

A brief summary of outcomes that can be gathered from Fig. 5, Fig. 6 is given hereinafter.

- the primary information gleaned from the utilisation of auxiliary parameters – notably A, C (inclusive of B's variability), D, and F (encompassing C and D) – is the observed non-sensitivity to the structure's height. This becomes evident by examining graphs plotted for $N_{st}$ (number of storeys) set equal to 2, 3, and 4;
- additionally, there is a notable absence of discernible influence stemming from variations in nominal yield strength, ranging from 300 to 500 MPa;
- lastly, it is crucial to emphasise that auxiliary parameters A, C, and F (with the exception of D) exhibit variations based on the displacement profile. Notably, parameter D remains constant irrespective of the system's ductility level.

It is also noteworthy that adopting mean parameters (A_m_, C_m_, D_m_, E_m_, and F_m_) does not result in significant errors in the transformation process. In this context, the metric for error is the non-dimensional relative variation calculated between the mean value and the analytical value. Even in the worst-case scenario, observed with A_m_, the error remains less than 5 %, even when considering a wide range of ductility values spanning from 0 to 20. This is attributed to the limited dispersion of values, maintaining accuracy even in the face of substantial variations in ductility.

In closing, it is noted that, although the DDBD-based procedure has been relatively unapplied to the precast concrete case up until now, the main significance of the above-discussed steps consists in providing readers/designers with a simple yet reliable and stable mechanics-based analytical/finite element model useable for a variety of applications including the derivation of inelastic performance-based spectra, with which this paper is chiefly concerned.

### 2.3 Incremental dynamic analysis

In order to execute IDA using the ESDOF system presented in Section 2.2.2, the set of structural parameters introduced above is varied within intervals of technical interest. The number of storeys ($N_{st}$) is modified ranging from one to four, and three different mass configurations (expressing $m_{top}$ as a percentage of $m_{i}$) are assumed. Additionally, changes in aspect ratio ($A_{r}$) and total height ($H_{n}$) are investigated. Account is also given of five distinct stability index values (θ). Although auxiliary parameter identification is executed for different steel grades (i.e. $f_{y}=$ 300, 400 and 500 MPa), the analyses presented here are exclusively focussed on the highest grade (i.e. $f_{y}=500$ MPa). Table 1, Table 2 show the variability of the structural parameters, as described earlier, for single- and multi-storey buildings, respectively.

**Table 1:** Variability in main structural parameters used for IDA for the single-storey frame case ($N_{st}=1$).

Stability index θ	Masses	Aspect ratio $A_{r}$	Total height $H_{n}$ [m]
0.05 0.10 0.15 0.20 0.30	$m_{top}$ = 100 % $m_{i}$ $m_{top}$ = 80 % $m_{i}$ $m_{top}$ = 60 % $m_{i}$	10 13 16 20	5 6 7 8 9 10 11 12

**Table 2:** Variability in main structural parameters used for IDA for the multi-storey frame case ($N_{st}=2,3,4$).

Stability index θ	Masses	Aspect ratio $A_{r}$	Interstorey height $H_{i}$ [m]
0.05 0.10 0.15 0.20 0.30	$m_{top}$ = 100 % $m_{i}$ $m_{top}$ = 80 % $m_{i}$ $m_{top}$ = 60 % $m_{i}$	10 13 16 20	3 3.5 4 4.5 5

**Fig. 7:**
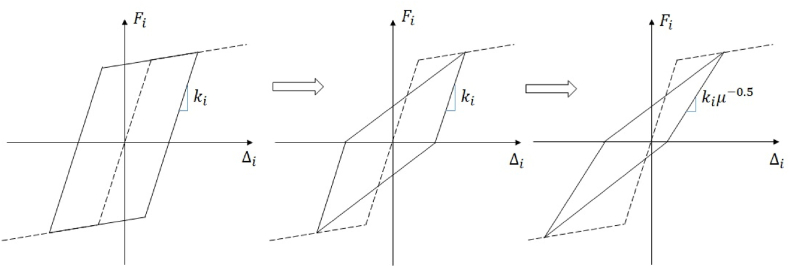
Constitutive material model: the classical elastic-plastic model (left), Clough hysteresis model (middle) and Takeda model (right) used in this research.

To undertake IDA, the nonlinear finite element software OpenSees [55] is employed. The nonlinear model is characterised by a rigid element with mass on top, and a nonlinear hinge positioned at the base. The hinge is tuned to emulate the behaviour of the ESDOF system, and the hysteretic rules incorporated into the numerical model are those of a classical Takeda model, as suggested in Priestley et al. [56]. The simplest model on the left in Fig. 7 resembles a bi-linear one; the Clough model improves on the elasto-plastic one by considering the degradation of the reloading stiffness, and the Takeda model improves on the Clough one by considering the unloading stiffness as a fraction of the elastic stiffness that degrades exponentially with increasing deformation ductility. In accordance with the modelling assumptions, it is important to note that no post-yield hardening is taken into account. Therefore, the maximum resisting moment at the base remains equal to $M_{\text{base}}$, regardless of the ductility demand. Additionally, as per the approach outlined by Adam and Jager [23,41], no material degradation is considered.

The ground motion records selected for carrying out IDA are carefully chosen to meet the following criteria.

• far-field conditions: the recording station's distance from the seismic event, measured using parameters like Joyner-Boore distance, closest to rupture distance, or epicenter distance, must fall within the range of 15–40 km;
• EC8 site conditions type C (180 m/s < $V_{s30}$ < 360 m/s);
• magnitude ranging from 5.0 to 8.0.

Hence, in the IDA, a total of 15 earthquake records are employed, and their primary characteristics are summarised in Table 3.

**Table 3:** Ground motion records selected to execute IDA.

Event	Station	$M_{w}//M_{l}//M_{s}$	Distance [km]	PGA [g]
Imperial Valley (1979)	6605 Delta	6.5//6.6//6.9	43.6 (Rrup)	0.351
Chi-Chi (1999)	CHY036	7.6//7.3//7.6	20.4 (Rrup)	0.294
Duzce (1999)	Bolu	7.1//7.2//7.3	17.6 (Rrup)	0.822
Livermore (1980)	Eastman Kodak	5.4//5.4//5.5	17.6 (Rrup)	0.301
Loma prieta (1989)	Gilroy Array#4	6.9//-//7.1	16.1 (Rrup)	0.417
Northridge (1994)	90053 Canega Park	6.7//6.6//6.7	15.8 (Rrup)	0.356
S. Fernando (1971)	135 LA-Hollywood	6.6//-//6.6	21.2 (Rrup)	0.174
Kocaeli (1999)	Iznik	7.4//-//7.8	31.8 (Rrup)	0.136
Whittier Narrows (1987)	90078 Compton	6.0//5.9//5.7	16.9 (Rrup)	0.333
Friuli, 2nd shock (1976)	Buia	5.6//-//5.8	19.5 (Repi)	0.233
Irpinia (1980)	Mercato S.Severino	6.90//-//-	29.8 (Rrup)	0.145
Val Comino (1984)	Cassino S.Elia	5.9//5.9//5.8	19.7 (Repi)	0.145
Umbria-Marche 1st shock (1997)	Castelnuovo (Assisi)	5.7//5.6//5.6	24.5 (Repi)	0.101
Umbria-Marche 2nd shock (1997)	Castelnuovo (Assisi)	6.0//5.8//6.1	21.5 (Repi)	0.172
L'Aquila (2009)	Avezzano	6.3//5.8//-	34.9 (Repi)	0.069

The outcome of IDA consists of a series of dynamic pushover curves, which are represented in dimensionless form using a relative intensity measure, $\left[S_{a}\left(T\right)/g\right]/\gamma$, that serves as a ductility-dependent response modification factor and that can simply be referred to as response modification factor; therein, $S_{a}$ is the normalised 5%-damped spectral acceleration at the structure's fundamental period T, and γ is defined as the ratio between the yield base shear and the total weight [23,24,41,57]. These curves, plotted against and the displacement ductility μ of the ESDOF system, are generated by progressively increasing the ground motion intensity until dynamic instability leading to collapse is reached. To follow nomenclature, this transition is reflected in Equation (26), which takes the form:

(26) $$R=\frac{S_{E}\left(T^{*}\right)\cdot m^{*}\cdot g\cdot H^{*}}{M_{\text{base}}}$$

where $S_{E}\left(T^{*}\right)$ represents the pseudo-acceleration spectrum ordinate at the natural elastic period of the ESDOF system for the provided records. Dynamic pushover curves are computed for increments of *R* value equal to 0.2.

## 3 Results and discussion

In this Section, analysis results are presented, focussing on limit states and drift spectra, while emphasising their dependence on key structural parameters. The breakdown of discussions is as follows.

- Section 3.1 presents examples of IDA curves, or dynamic pushover curves, corresponding to few ground motion records and inelastic spectra with statistics;
- Section 3.2 encompasses diagrams depicting the median inelastic spectra with varying aspect ratio ($A_{r}$);
- Section 3.3 illustrates diagrams showcasing the median inelastic spectra with varying mass configuration ($m_{top}$ = % $m_{i}$);
- Section 3.4 showcases diagrams of median inelastic spectra for variations in the stability index (θ).

In Section 3.1, dynamic pushover curves are presented in dimensionless form, accompanied by selected examples of inelastic spectra, and are briefly commented upon. This approach ensures that the graphical representations serve a dual purpose: being initially introduced for contextual understanding and being subsequently elaborated upon in later Sections (from 3.2 to 3.4) to elucidate their significance within specific contexts. To further facilitate the practical application of these diagrams as design tools, data regression is also performed, carefully considering the specific parameter variability associated with each spectrum.

### 3.1 Dynamic pushover curves and inelastic spectra

**Fig. 8:**
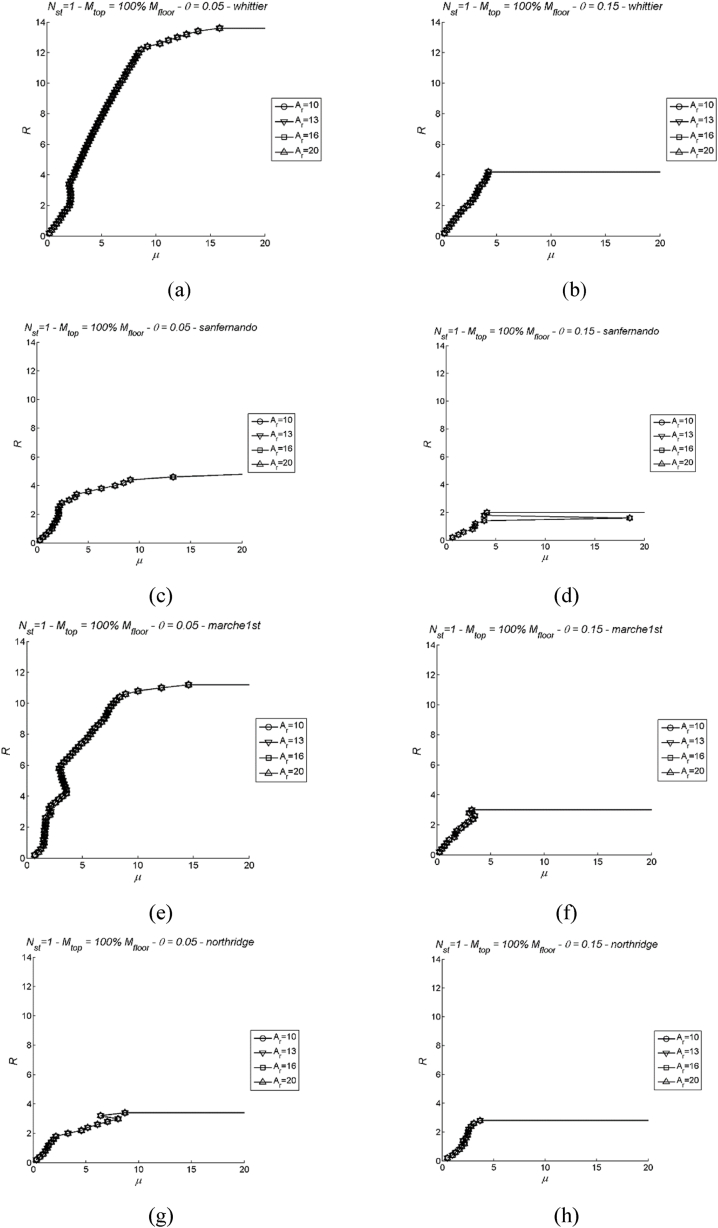
Examples of IDA curves for single-storey precast structures; two values of stability index θ = 0.05 (left) and 0.15 (right), four records (Whittier Narrows, S. Fernando, Umbria-Marche 1st, Northridge), $A_{r}=$ 10, 13, 16 and 20, and $m_{top}$ = 100 % $m_{i}$. More specifically, (a) stability index θ = 0.05 and earthquake Whittier Narrows; (b) stability index θ = 0.15 and earthquake Whittier Narrows; (c) stability index θ = 0.05 and earthquake S. Fernando; (d) stability index θ = 0.15 and earthquake S. Fernando; (e) stability index θ = 0.05 and earthquake Umbria-Marche 1st; (f) stability index θ = 0.15 and earthquake Umbria-Marche 1st; (g) stability index θ = 0.05 and earthquake Northridge; (h) stability index θ = 0.15 and earthquake Northridge.

**Fig. 9:**
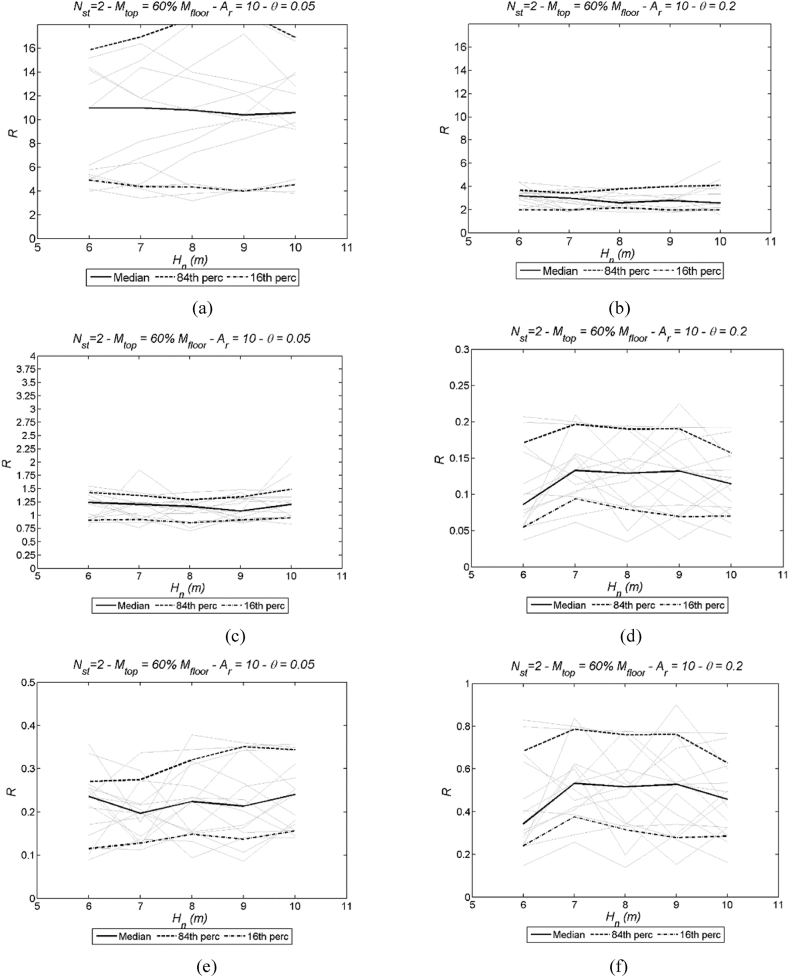
Examples of: (a) LSS at collapse limit state (CC-LS3) and (b) LSS at damage limit state (DL-LS2); (c) LSS at serviceability limit state (SL-LS1) and (d) DLS at 0.5 % drift limit (DR05); (e) DLS at 1.0 % limit (DR10) and (f) DLS at 2.0 % drift limit (DR20).

Dynamic pushover curves are presented here in dimensionless form by correlating the response modification factor – see Equation (26) – with the ESDOF system ductility μ. Examples of these curves are given in Fig. 8 corresponding to: number of storeys $N_{st}=1$, $A_{r}=$ 10, 13, 16 and 20, two values of stability index θ = 0.05 and 0.15, four ground motion records according to Table 3 (Whittier Narrows, S. Fernando, Umbria-Marche 1st, Northridge), and $m_{top}$ = 100 % $m_{i}$. It is evident from Fig. 8 that the dynamic pushover curves are not influenced by the aspect ratio $A_{r}$, as no significant variation can be discerned. Limit states and the attainment of drift limits are identified through the ESDOF system ductility parameter, denoted as $\mu_{\lim}$. This parameter can be readily determined using Equations (7), (8), (18) [58], depending on whether drift limit or strain limit is imposed. It is important to note that the collapse limit state is identified through the procedure outlined above. To generate inelastic spectra for each limit state and drift limit, corresponding response modification factor values are plotted for each ground motion record and structure's height. Examples of these diagrams are illustrated in Fig. 9 in case of: number of storeys $N_{st}=2$, two values of the stability index θ = 0.05, and 0.2, $m_{top}$ = 60 % $m_{i}$, $A_{r}$ = 10; in case of DLS, three drift limits are depicted corresponding to DR05, DR10 and DR20. In addition to the median value, the 16th and the 84th percentiles serve as a measure of dispersion. These three key metrics are thoughtfully portrayed in Fig. 9, offering a visual representation of the variability inherently related to the response modification factor (see Fig. 9a–f). This representation not only captures the central tendency of the data through the median but also provides insight into the spread of outcomes by showcasing the 16th and 84th percentiles.

In the subsequent three sub-sections, detailed investigation and commentary are provided for these graphs, delving into their variability concerning aspect ratio, mass configuration, and stability index. Such discussions aim at offering a comprehensive understanding of how these key structural parameters influence the observed variations.

### 3.2 Inelastic spectra variability with aspect ratio

**Fig. 10:**
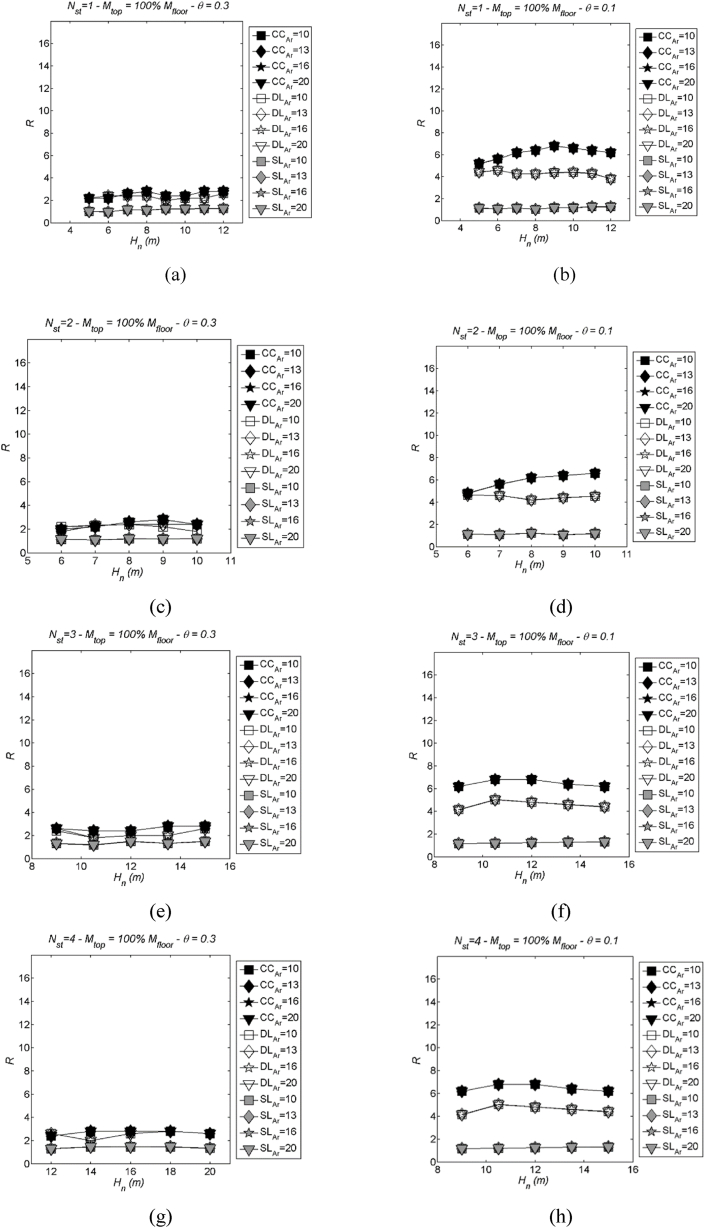
Examples of LSS with varying aspect ratio ($A_{r}$): number of storeys $N_{st}=$ 1, 2, 3 and 4, stability index θ = 0.3 (left) and θ = 0.1 (right), $m_{top}$ = 100 % $m_{i}$. More specifically, (a) $N_{st}=$ 1 and θ = 0.3; (b) $N_{st}=$ 1 and θ = 0.1; (c) $N_{st}=$ 2 and θ = 0.3; (d) $N_{st}=$ 2 and θ = 0.1; (e) $N_{st}=$ 3 and θ = 0.3; (f) $N_{st}=$ 3 and θ = 0.1; (g) $N_{st}=$ 4 and θ = 0.3; (h) $N_{st}=$ 4 and θ = 0.1.

**Fig. 11:**
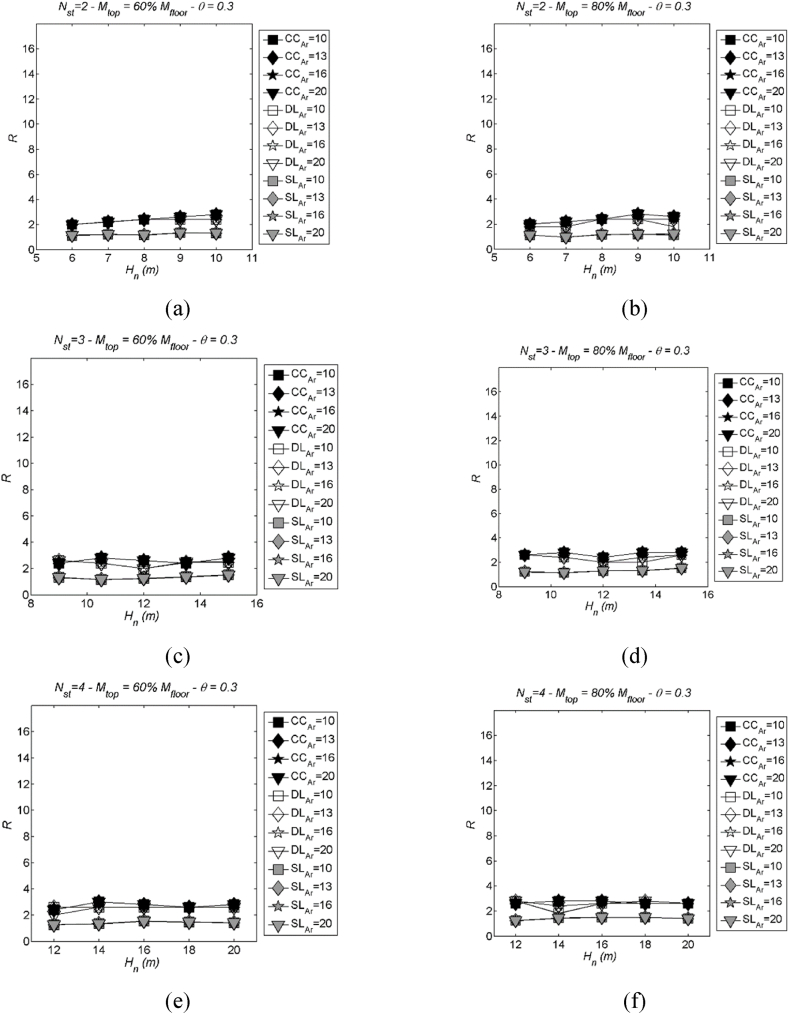
Examples of LSS with varying aspect ratio ($A_{r}$): three values of storeys $N_{st}=$ 2, 3 and 4 in case of two values of mass $m_{top}$ = 60 % (left) and 80 % $m_{i}$ (right), stability index θ = 0.3. More specifically, (a) $N_{st}=$ 2 and $m_{top}$ = 60 % $m_{i}$; (b) $N_{st}=$ 2 and $m_{top}$ = 80 % $m_{i}$; (c) $N_{st}=$ 3 and $m_{top}$ = 60 % $m_{i}$; (d) $N_{st}=$ 3 and $m_{top}$ = 80 % $m_{i}$; (e) $N_{st}=$ 4 and $m_{top}$ = 60 % $m_{i}$; (f) $N_{st}=$ 4 and $m_{top}$ = 80 % $m_{i}$.

Diagrams depicting the median inelastic spectra with varying aspect ratio ($A_{r}$) are provided in Fig. 10, Fig. 11. Fig. 10(left) shows the results in case of: number of storeys $N_{st}=$ 1, 2, 3 and 4, stability index θ = 0.3, $m_{top}$ = 100 % $m_{i}$; Fig. 11 shows three values of storeys $N_{st}=$ 2, 3 and 4 in case of two values of mass $m_{top}$ = 60 % and 80 % $m_{i}$, stability index θ = 0.3. Fig. 10 (right) is again an example to show the same trend, compared to Fig. 10 (left), simply by changing stability index to θ = 0.1 and $m_{top}$ = 100 % $m_{i}$, $N_{st}=$ 1, 2, 3 and 4.

As depicted in Fig. 10, Fig. 11, the LSS displays negligible variability in relation to $A_{r}$, maintaining closely aligned values across the specified range of aspect ratios ($A_{r}$ ranging from 10 to 20, as outlined in Table 1, Table 2). This consistent behaviour can be attributed to the observed stability in the dynamic pushover curves, illustrated in Fig. 8, which remain relatively constant as $A_{r}$ changes.

**Fig. 12:**
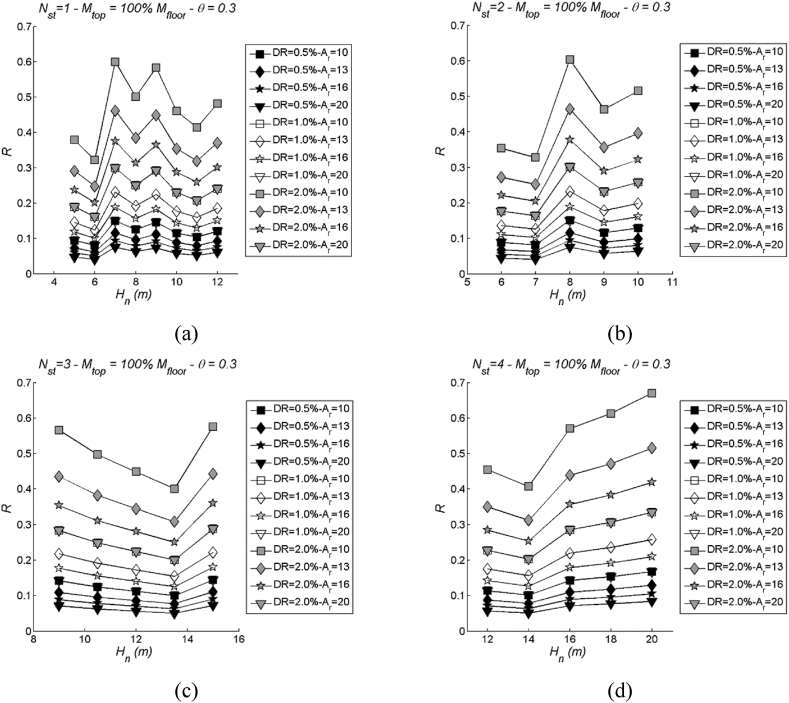
Examples of DLS with varying aspect ratio ($A_{r}$): number of storeys $N_{st}=$ 1, 2, 3 and 4, stability index θ = 0.3 and $m_{top}$ = 100 % $m_{i}$. More specifically, (a) $N_{st}=$ 1; (b) $N_{st}=$ 2; (c) $N_{st}=$ 3; (d) $N_{st}=$ 4.

**Fig. 13:**
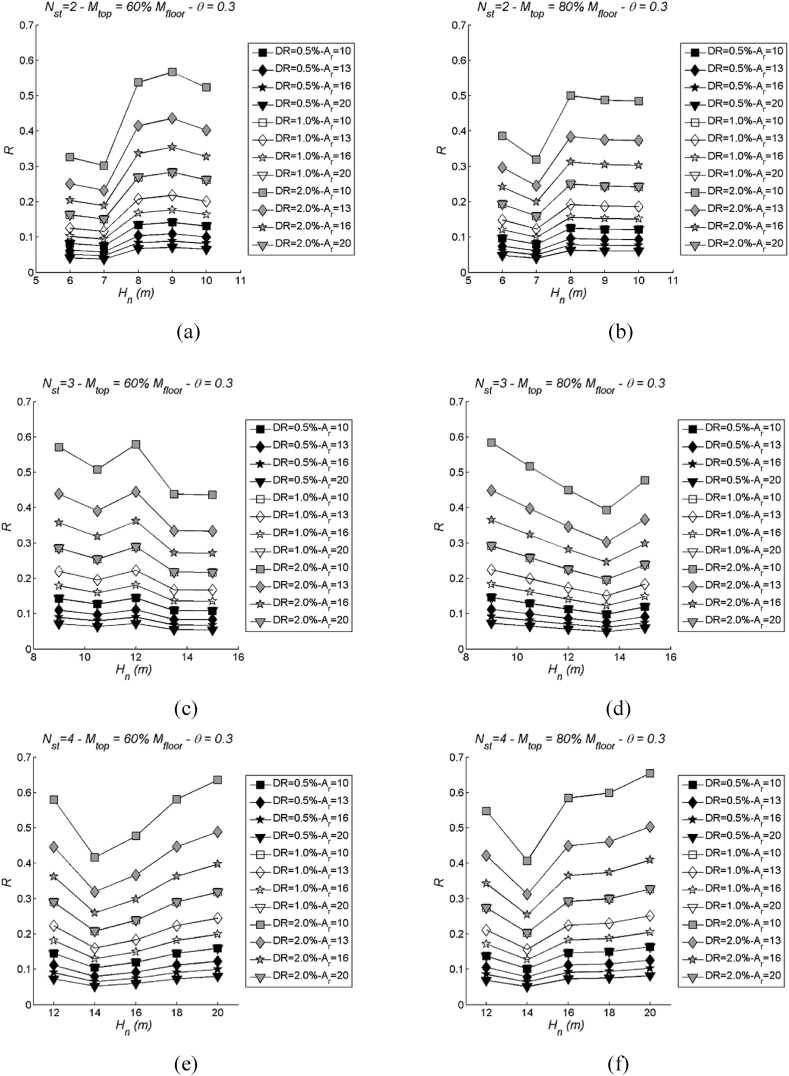
Examples of DLS with varying aspect ratio ($A_{r}$): three values of storeys $N_{st}=$ 2, 3 and 4 in case of two values of mass $m_{top}$ = 60 % (left) and 80 % $m_{i}$ (right), stability index θ = 0.3. More specifically, (a) $N_{st}=$ 2 and $m_{top}$ = 60 % $m_{i}$; (b) $N_{st}=$ 2 and $m_{top}$ = 80 % $m_{i}$; (c) $N_{st}=$ 3 and $m_{top}$ = 60 % $m_{i}$; (d) $N_{st}=$ 3 and $m_{top}$ = 80 % $m_{i}$; (e) $N_{st}=$ 4 and $m_{top}$ = 60 % $m_{i}$; (f) $N_{st}=$ 4 and $m_{top}$ = 80 % $m_{i}$.

Conversely, the DLS demonstrates substantial variation with changes in $A_{r}$, revealing a discernible pattern for all considered drift limits (Fig. 12, Fig. 13 employ the same parameters as Fig. 10, Fig. 11, respectively, for comparison). The degree of variation becomes more pronounced as the drift limit increases. This observed trend can be elucidated by examining the formulation of the effective ductility limit, $\mu_{\lim}$. It can be demonstrated that, when imposing a strain limit curvature in the form of $\Phi_{\lim}=\frac{k}{h}$, this effectively eliminates the aspect ratio $A_{r}$ from the $\mu_{\lim}$ equation. On the other hand, the effective ductility corresponding to a drift limit retains its dependency on the aspect ratio. This clarification helps elucidate why the DLS exhibits more significant variation with changes in $A_{r}$ compared to the LSS.

### 3.3 Inelastic spectra variability with mass configuration

**Fig. 14:**
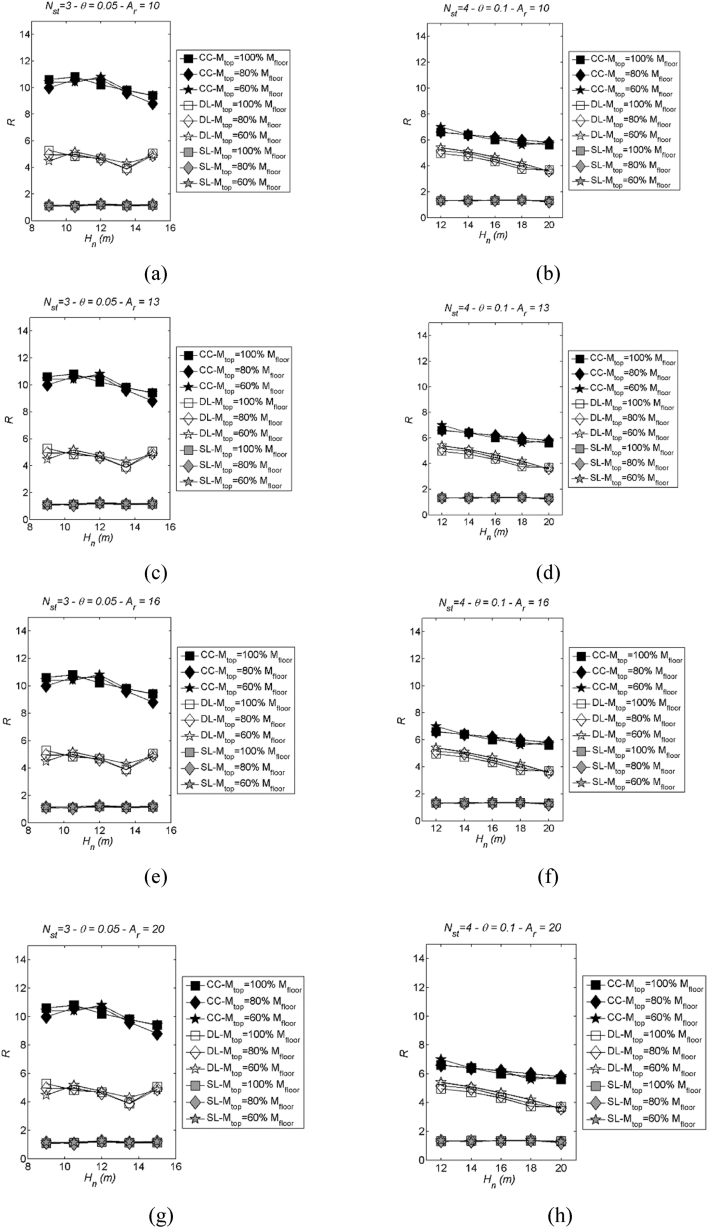
Examples of LSS to show variability with mass configuration: two values of storeys $N_{st}=$ 3 and 4 in case of stability index θ = 0.05 (left) and 0.1 (right); $A_{r}$ = 10, 13, 16 and 20. More specifically, (a) $N_{st}=$ 3, θ = 0.05 and $A_{r}$ = 10; (b) $N_{st}=$ 4, θ = 0.1 and $A_{r}$ = 10; (c) $N_{st}=$ 3, θ = 0.05 and $A_{r}$ = 13; (d) $N_{st}=$ 4, θ = 0.1 and $A_{r}$ = 13; (e) $N_{st}=$ 3, θ = 0.05 and $A_{r}$ = 16; (f) $N_{st}=$ 4, θ = 0.1 and $A_{r}$ = 16; (g) $N_{st}=$ 3, θ = 0.05 and $A_{r}$ = 20; (h) $N_{st}=$ 4, θ = 0.1 and $A_{r}$ = 20.

**Fig. 15:**
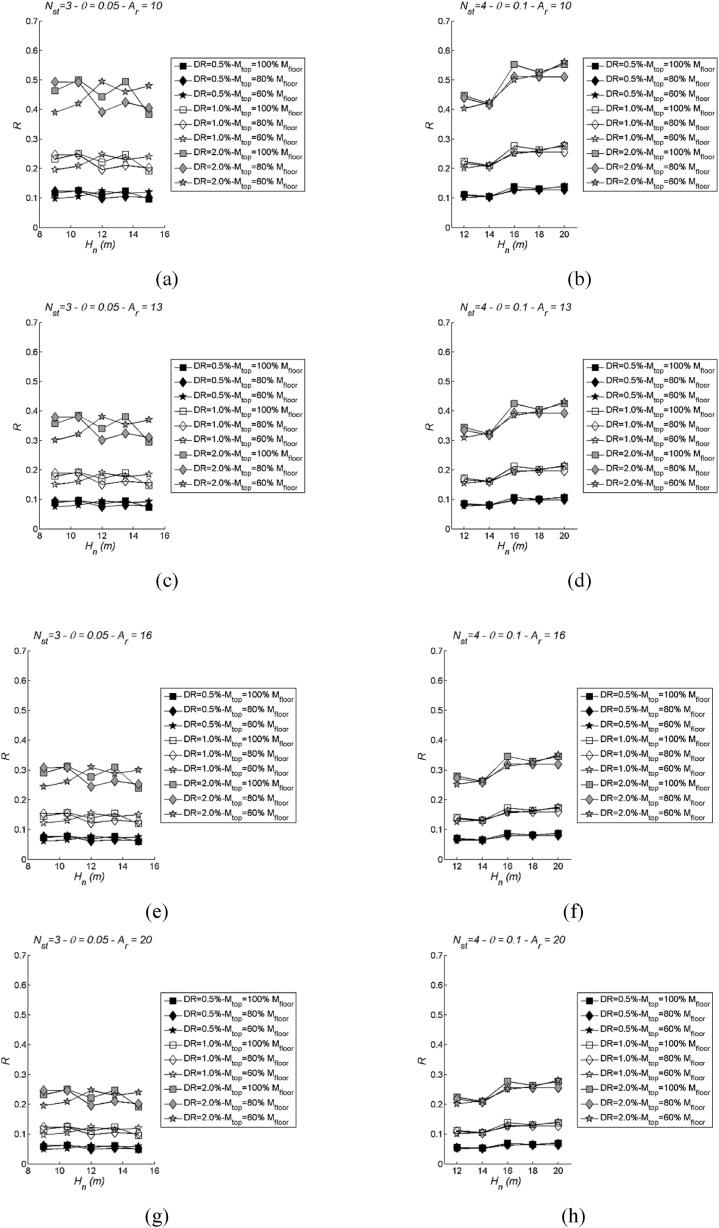
Examples of DLS to show variability with mass configuration: two values of storeys $N_{st}=$ 3 and 4 in case of stability index θ = 0.05 (left) and 0.1 (right); $A_{r}$ = 10, 13, 16 and 20. More specifically, (a) $N_{st}=$ 3, θ = 0.05 and $A_{r}$ = 10; (b) $N_{st}=$ 4, θ = 0.1 and $A_{r}$ = 10; (c) $N_{st}=$ 3, θ = 0.05 and $A_{r}$ = 13; (d) $N_{st}=$ 4, θ = 0.1 and $A_{r}$ = 13; (e) $N_{st}=$ 3, θ = 0.05 and $A_{r}$ = 16; (f) $N_{st}=$ 4, θ = 0.1 and $A_{r}$ = 16; (g) $N_{st}=$ 3, θ = 0.05 and $A_{r}$ = 20; (h) $N_{st}=$ 4, θ = 0.1 and $A_{r}$ = 20.

The diagrams illustrating the median inelastic spectra with varying mass configurations are presented in Fig. 14, Fig. 15, which pertain to the LSS and the DLS, respectively. In both cases, the following parameters have been plotted: $N_{st}=$ 3 and 4, stability index θ = 0.05 and 0.1 and $A_{r}$ = 10, 13, 16 and 20. Both LSS and DLS exhibit small variability concerning mass configuration. The variations in comparison to the base case (where $m_{top}$ = 100 % $m_{i}$) are negligibly small at the CC-LS3, DL-LS2, and SL-LS1, with slightly greater deviations as the response modification factor increases (i.e. transitioning from SL-LS1 to CC-LS3). DLS appears to be more sensitive to changes in roof mass configuration. However, the differences observed are limited and tend to decrease as the response modification factor decreases.

### 3.4 Inelastic spectra variability with stability index

**Fig. 16:**
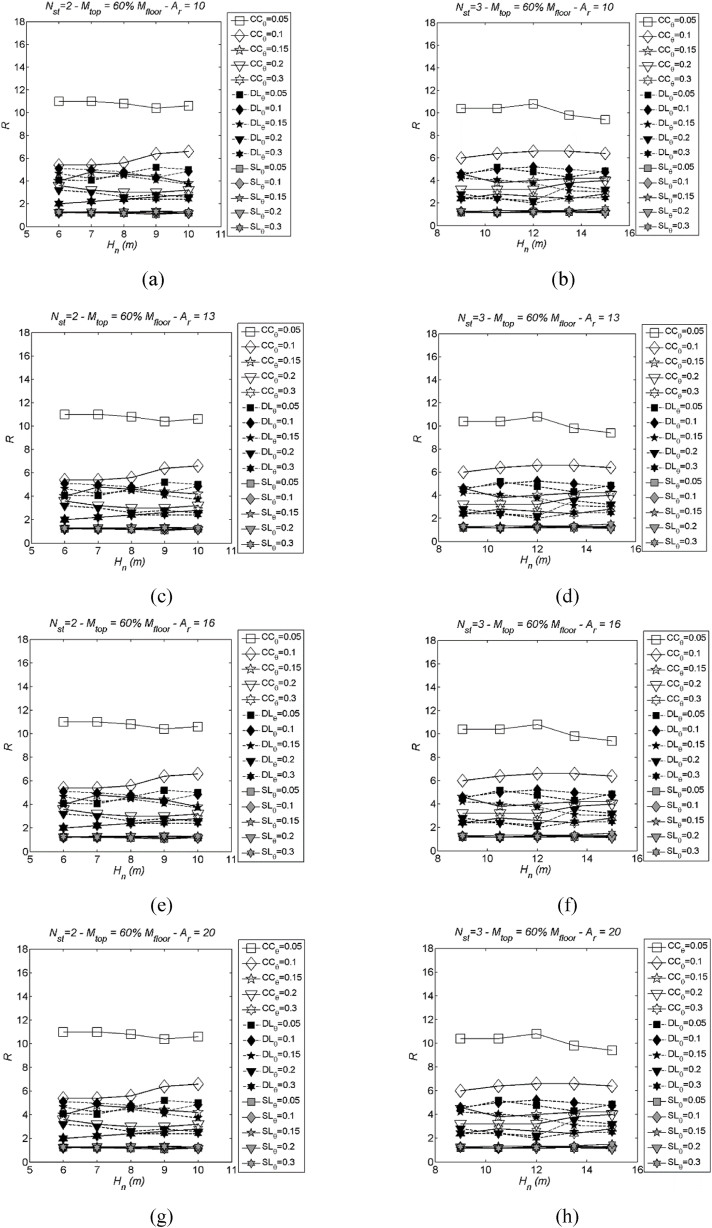
Examples of LSS to show variability with the stability index: two values of storeys $N_{st}=$ 2 (left) and 3 (right) in case of $m_{top}$ = 60 % $m_{i}$; $A_{r}$ = 10, 13, 16 and 20. More specifically, (a) $N_{st}=$ 2 and $A_{r}$ = 10; (b) $N_{st}=$ 3 $A_{r}$ = 10; (c) $N_{st}=$ 2 and $A_{r}$ = 13; (d) $N_{st}=$ 3 and $A_{r}$ = 13; (e) $N_{st}=$ 2 and $A_{r}$ = 16; (f) $N_{st}=$ 3 and $A_{r}$ = 16; (g) $N_{st}=$ 2 and $A_{r}$ = 20; (h) $N_{st}=$ 3 and $A_{r}$ = 20.

**Fig. 17:**
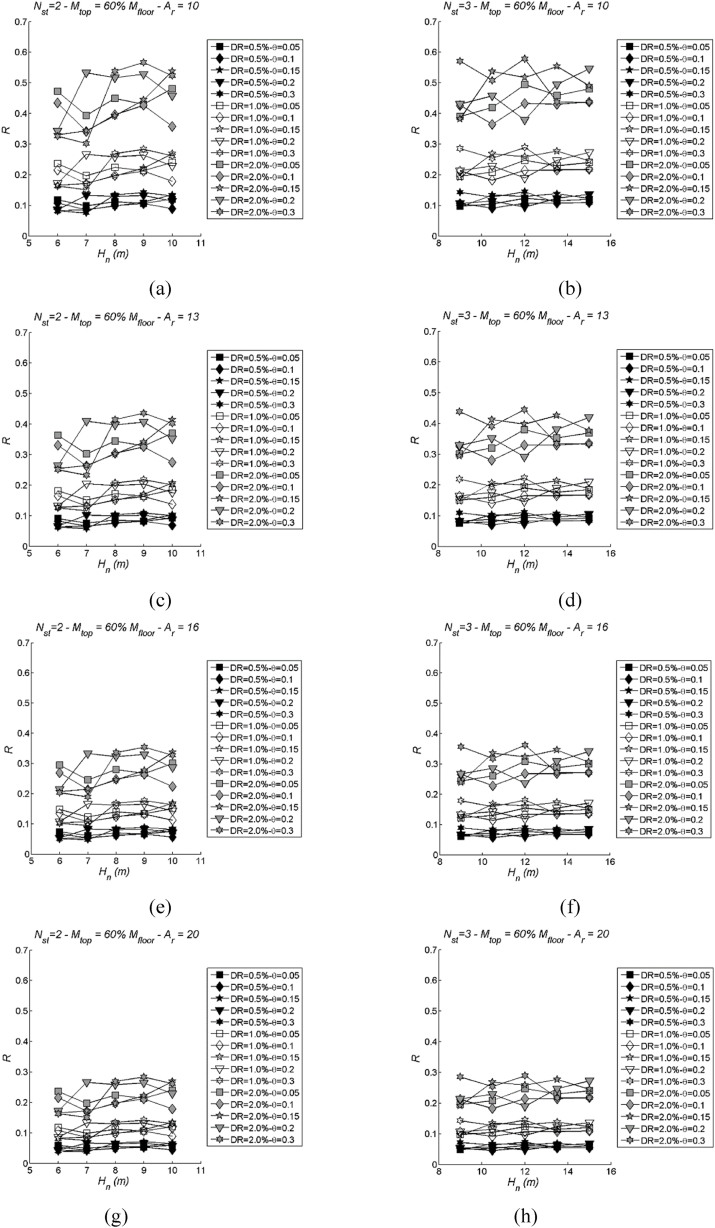
Examples of DLS to show variability with the stability index: two values of storeys $N_{st}=$ 2 (left) and 3 (right) in case of $m_{top}$ = 60 % $m_{i}$; $A_{r}$ = 10, 13, 16 and 20. More specifically, (a) $N_{st}=$ 2 and $A_{r}$ = 10; (b) $N_{st}=$ 3 $A_{r}$ = 10; (c) $N_{st}=$ 2 and $A_{r}$ = 13; (d) $N_{st}=$ 3 and $A_{r}$ = 13; (e) $N_{st}=$ 2 and $A_{r}$ = 16; (f) $N_{st}=$ 3 and $A_{r}$ = 16; (g) $N_{st}=$ 2 and $A_{r}$ = 20; (h) $N_{st}=$ 3 and $A_{r}$ = 20.

Fig. 16, Fig. 17 also provide insights into the trends of median inelastic spectra with varying stability index. The LSS (Fig. 16) appears to be strongly influenced by this parameter, demonstrating an increasing dependence as transitions are made from SL-LS1 to the CC-LS3. This suggests that the stability index has a notable impact on LSS behaviour.

Conversely, the DLS (Fig. 17) does not exhibit a clear, discernible pattern in response to stability index variations. The results seem to scatter around mean values at each drift limit, and this scattering becomes more pronounced as the drift limit increases. This suggests that the influence of the stability index on DLS is less pronounced and less predictable compared to its effect on LSS.

### 3.5 Regression of median LSS

In this sub-section, a regression is proposed for the median LSS. As previously mentioned, these results are not affected by the aspect ratio ($A_{r}$). Henceforth, the regression analysis exclusively incorporates the structure height ($H_{n}$) and the stability index (θ) as parameters. The number of storeys ($N_{st}$) is intentionally omitted as a regression parameter due to its observed impact on the response modification factor resulting in negligible variations. To simplify the interpretation of analysis results, spectra for different $N_{st}$ are presented together, and $H_{n}$ is assumed as the sole building height-related parameter. The regression is conducted using a polynomial function with the ordinary least squares method. The quality of the approximation is assessed using the coefficient of determination $R^{2}$, calculated as follows:

(27) $$R^{2}=\frac{RSS}{SYY}$$

where RSS refers to the sum of squared residuals concerning the mean value of the regression surface, while SYY represents the sum of squared residuals concerning the mean of the response, as explained in Weisberg [59]. A coefficient of determination ($R^{2}$) equal to 1 indicates a perfect alignment between the regression surface and the data, signifying that the model precisely predicts the observed outcomes. When $R^{2}$ is greater than 0 but less than 1, it suggests that the mean regression surface offers a better fit to the data than their mean value alone. However, if $R^{2}$ is less than 1, it implies that the regression approximation is less accurate than simply using the mean value to describe the data. In such cases, the model may not fully capture the variability present in the dataset, and additional factors may need to be considered for a more accurate representation.

Additional valuable information about the quality of the fitting can be obtained from the residual mean square (RMS), denoted as $\sigma^{2}$. This metric offers an estimate of the variance of the prediction and is calculated as follows:

(28) $$\sigma^{2}=\frac{RSS}{n_{df}}$$

where $n_{df}$ represents the residual degree of freedom, which is essentially the number of cases minus the parameter count of the mean function. Indeed, the standard error of regression (STE) is derived from the RMS and is calculated as the square root of RMS:

(29) $$STE=\sqrt{\frac{RSS}{n_{df}}}$$

The model identification procedure is conducted with the aim of maximising the coefficient of determination ($R^{2}$) while using the minimum number of parameters. Below, the mean function and regression parameters are provided for each spectrum (CC-LS3, DL-LS2, and SL-LS1). Additionally, the regression indices are retrieved and discussed to assess the quality and validity of the models.

**Fig. 18:**
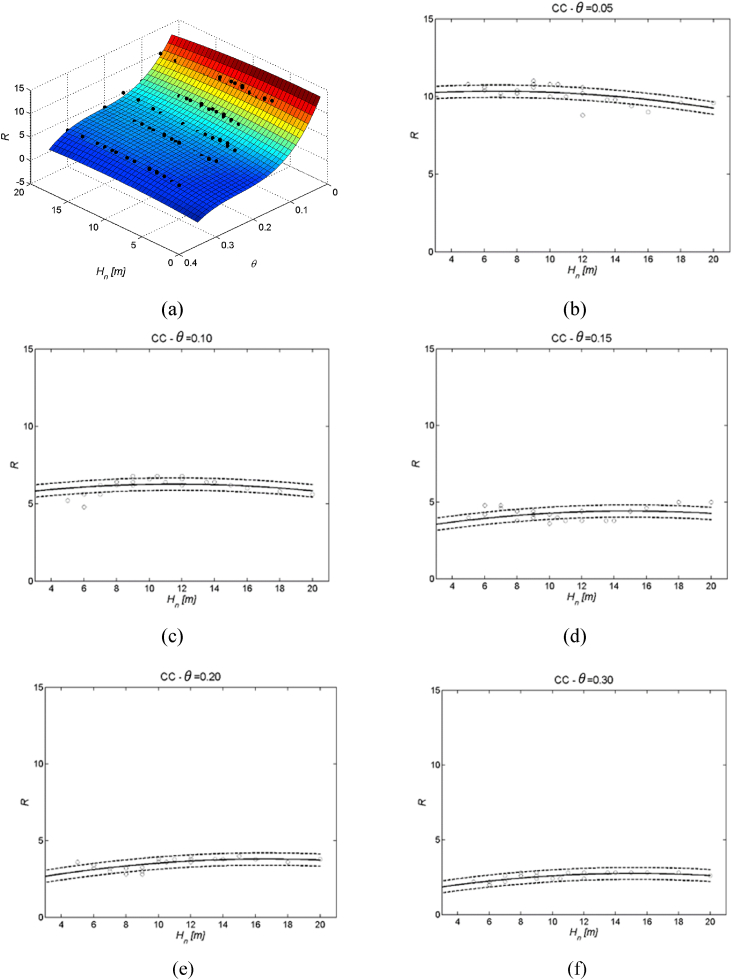
Regression for CC-LS3: mean surface (top left) as a function of $H_{n}$, R, and θ; single curves corresponding to θ= 0.05, 0.10, 0.15, 0.20, and 0.30. More specifically, (a) mean surface; (b) R versus $H_{n}$ for θ= 0.05; (c) R versus $H_{n}$ for θ= 0.10; (d) R versus $H_{n}$ for θ= 0.15; (e) R versus $H_{n}$ for θ= 0.20; (f) R versus $H_{n}$ for θ= 0.30.

**Fig. 19:**
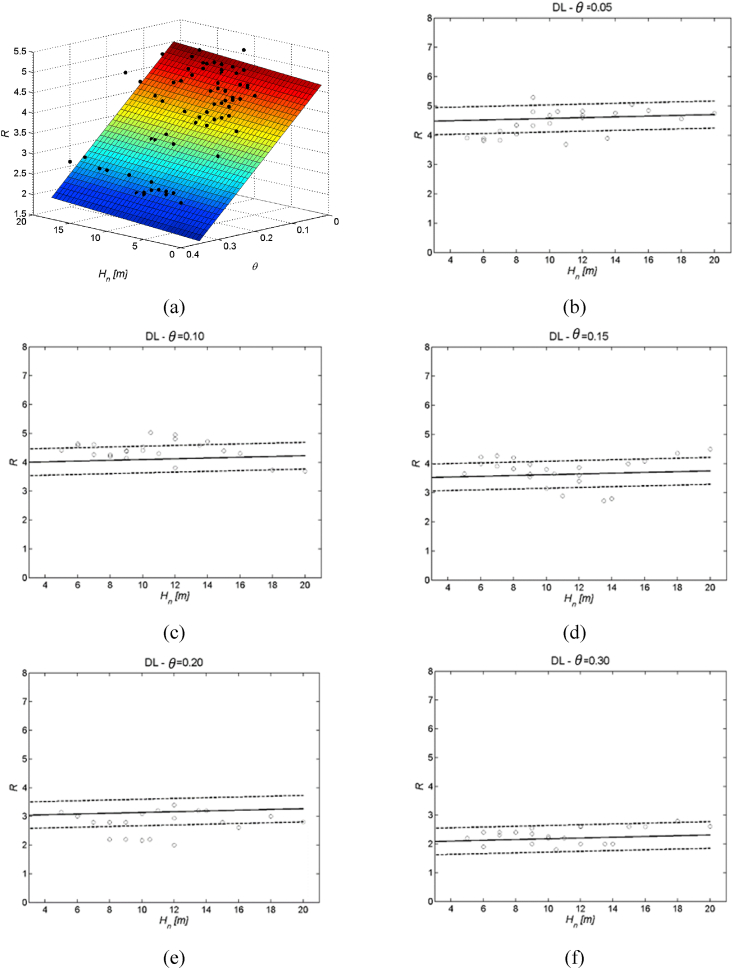
Regression for DL-LS2: mean surface (top left) as a function of $H_{n}$, R, and θ; single curves corresponding to θ= 0.05, 0.10, 0.15, 0.20, and 0.30. More specifically, (a) mean surface; (b) R versus $H_{n}$ for θ= 0.05; (c) R versus $H_{n}$ for θ= 0.10; (d) R versus $H_{n}$ for θ= 0.15; (e) R versus $H_{n}$ for θ= 0.20; (f) R versus $H_{n}$ for θ= 0.30.

**Fig. 20:**
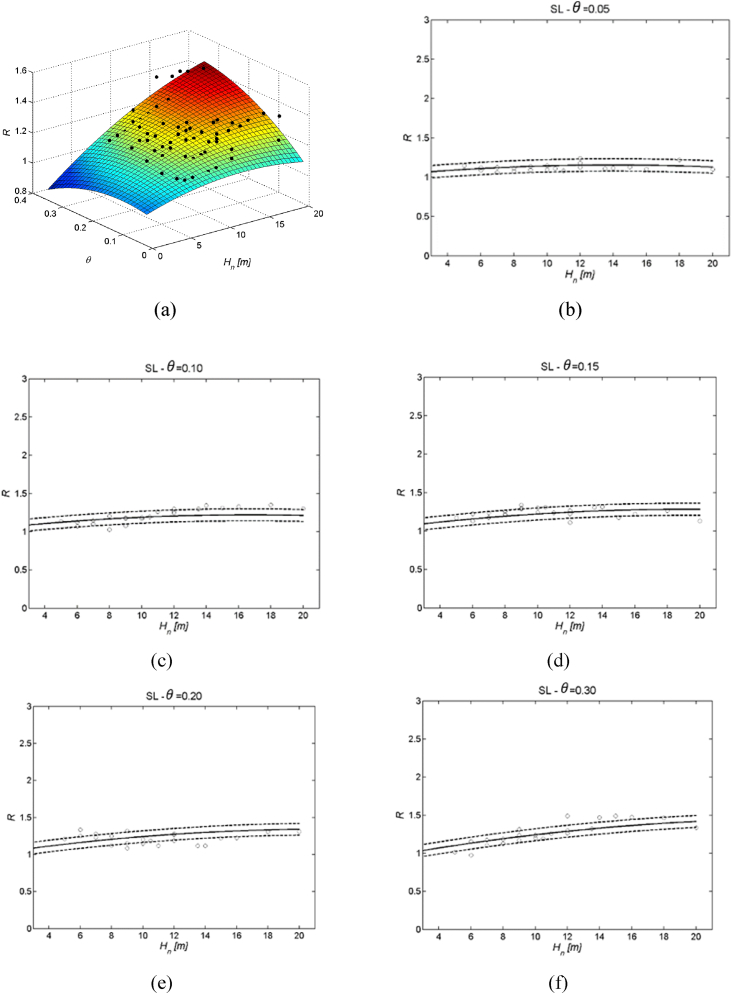
Regression for SL-LS1: mean surface (top left) as a function of $H_{n}$, R, and θ; single curves corresponding to θ= 0.05, 0.10, 0.15, 0.20, and 0.30. More specifically, (a) mean surface; (b) R versus $H_{n}$ for θ= 0.05; (c) R versus $H_{n}$ for θ= 0.10; (d) R versus $H_{n}$ for θ= 0.15; (e) R versus $H_{n}$ for θ= 0.20; (f) R versus $H_{n}$ for θ= 0.30.

(a) Collapse Limit Spectra – CC-LS3 (Fig. 18). The mean function of CC spectra is given by $\bar{R}\left(H_{n},\theta \right)=p_{00}+p_{01}\theta +p_{20}H_{n}^{2}+p_{11}H_{n}\theta +p_{02}\theta^{2}+p_{12}H_{n}\theta^{2}+p_{03}\theta^{3}$. The mean values of the regression coefficients ($p_{ii}$) along with their corresponding 95 % confidence intervals (${CI}_{95}$) are presented in Table 4. Additionally, the regression indices can be found in Table 5.
(b) Damage Limit Spectra – DL-LS2 (Fig. 19). The mean function of DL spectra is given by $\bar{R}\left(H_{n},\theta \right)=p_{00}+p_{01}\theta +p_{10}H_{n}$. The mean values of the regression coefficients ($p_{ii}$) along with their corresponding 95 % confidence intervals (${CI}_{95}$) are presented in Table 6. Additionally, the regression indices can be found in Table 7.
(c) Serviceability Limit Spectra – SL-LS1 (Fig. 20). The mean function of SL spectra is given by $\bar{R}\left(H_{n},\theta \right)=p_{00}+p_{01}\theta +p_{10}H_{n}+p_{02}\theta^{2}+p_{20}H_{n}^{2}+p_{11}H_{n}\theta$. The mean values of the regression coefficients ($p_{ii}$) along with their corresponding 95 % confidence intervals (${CI}_{95}$) are presented in Table 8. Additionally, the regression indices can be found in Table 9.

### 3.6 Regression of median DLS

Herein, a regression is proposed for the median DLS. As previously observed, these spectra are primarily influenced by the structural parameters $H_{n}$ (building height) and $A_{r}$ (aspect ratio), while variations in the stability index θ do not reveal clear patterns. Furthermore, the point made regarding the influence of the number of storeys ($N_{st}$) in the previous sub-section remains applicable in this case too. Based on these considerations, regression is executed using the same procedures as employed with the LSS, utilising the two structural parameters $H_{n}$ and $A_{r}$.

**Fig. 21:**
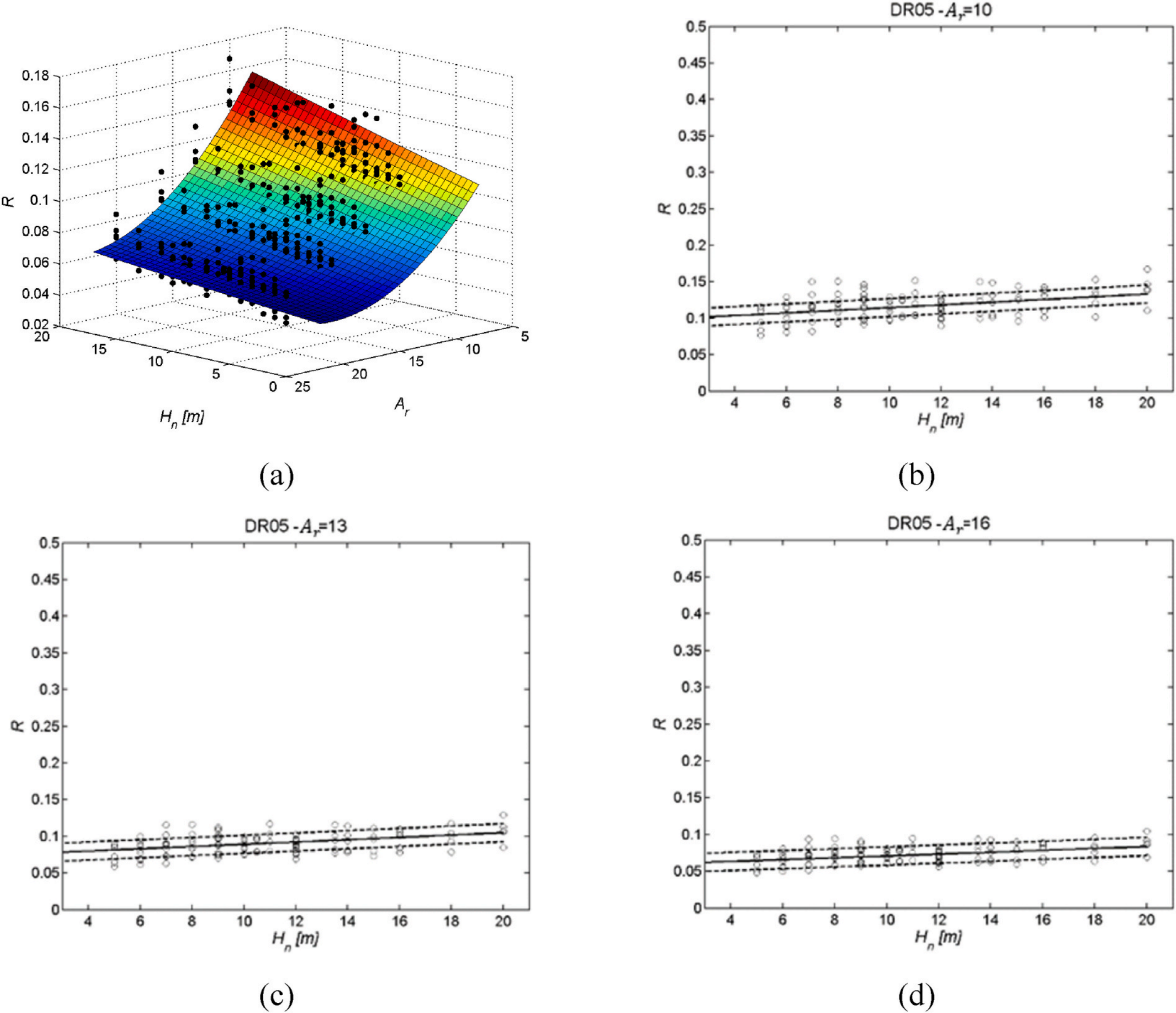
Regression for DR05: mean surface (top left) as a function of $H_{n}$, R, and $A_{r}$; single curves corresponding to $A_{r}=$ 10, 13, and 16. More specifically, (a) mean surface; (b) R versus $H_{n}$ for $A_{r}=$ 10; (c) R versus $H_{n}$ for $A_{r}=$ 13; (d) R versus $H_{n}$ for $A_{r}=$ 16.

**Fig. 22:**
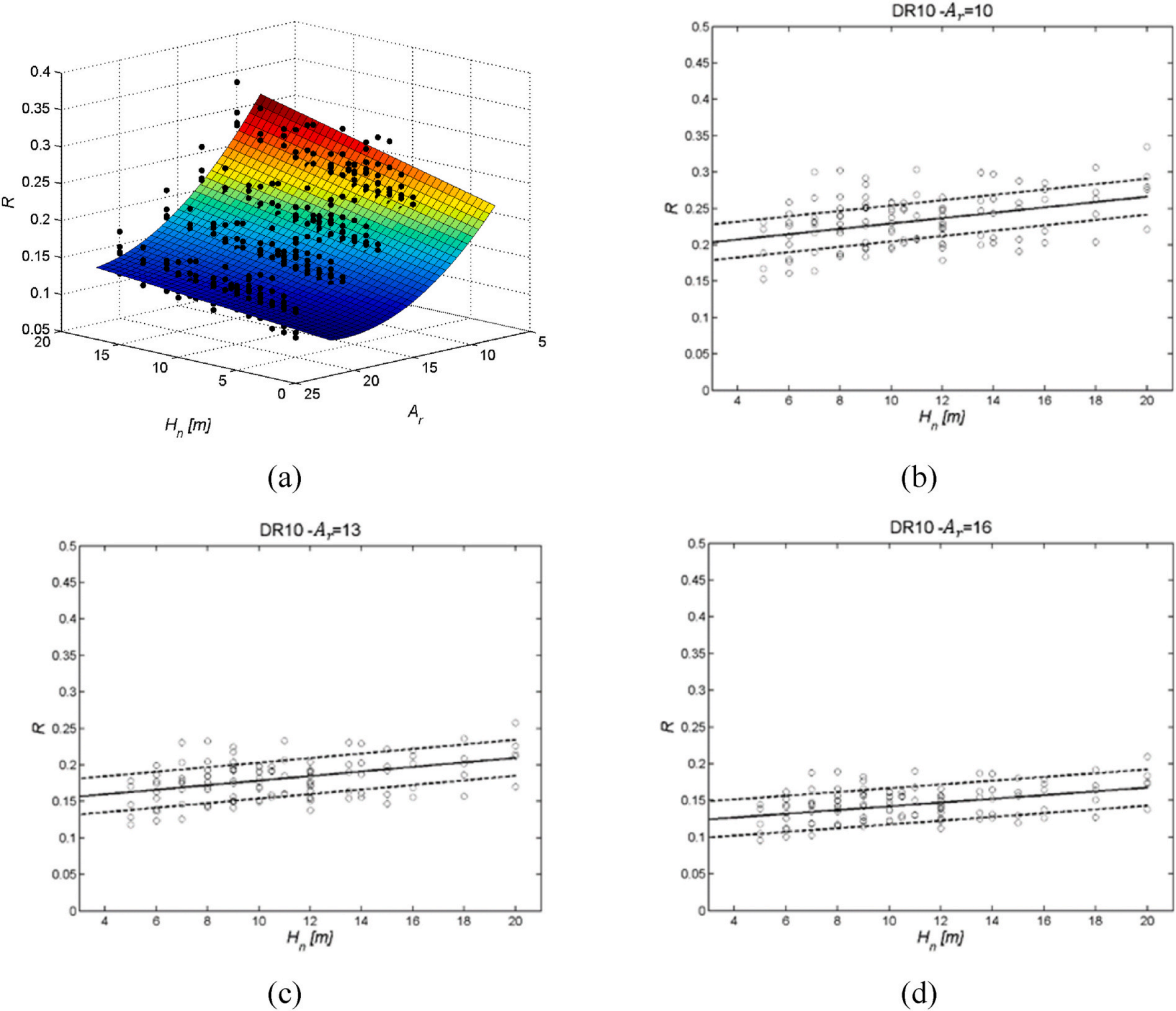
Regression for DR10: mean surface (top left) as a function of $H_{n}$, R, and $A_{r}$; single curves corresponding to $A_{r}=$ 10, 13, and 16. More specifically, (a) mean surface; (b) R versus $H_{n}$ for $A_{r}=$ 10; (c) R versus $H_{n}$ for $A_{r}=$ 13; (d) R versus $H_{n}$ for $A_{r}=$ 16.

(a) 0.5 % Drift limit spectra – DR05 (Fig. 21). The mean function of DR05 spectra is given by $\bar{R}\left(H_{n},A_{r}\right)=p_{00}+p_{01}A_{r}+p_{10}H_{n}+p_{02}A_{r}^{2}+p_{11}H_{n}A_{r}$. The mean values of the regression coefficients ($p_{ii}$) along with their corresponding 95 % confidence intervals (${CI}_{95}$) are presented in Table 10. Additionally, the regression indices can be found in Table 11.
(b) 1.0 % Drift limit spectra – DR10 (Fig. 22). The mean function of DR10 spectra is given by $\bar{R}\left(H_{n},A_{r}\right)=p_{00}+p_{01}A_{r}+p_{10}H_{n}+p_{02}A_{r}^{2}+p_{11}H_{n}A_{r}$. The mean values of the regression coefficients ($p_{ii}$) along with their corresponding 95 % confidence intervals (${CI}_{95}$) are presented in Table 12. Additionally, the regression indices can be found in Table 13.
(c) 2.0 % Drift limit spectra – DR20 (Fig. 23). The mean function of DR20 spectra is given by $\bar{R}\left(H_{n},A_{r}\right)=p_{00}+p_{01}A_{r}+p_{10}H_{n}+p_{02}A_{r}^{2}+p_{11}H_{n}A_{r}$. The mean values of the regression coefficients ($p_{ii}$) along with their corresponding 95 % confidence intervals (${CI}_{95}$) are presented in Table 14. Additionally, the regression indices can be found in Table 15.

## 4 Conclusions

The objective of this study is to develop a simplified method for evaluating second-order effects in precast reinforced concrete frames featuring pinned beam-column connections when subjected to earthquake-induced actions. This particular class of structures is highly vulnerable to such effects due to their inherent lateral deformability and, most regrettably, this vulnerability was illustrated by the significant damage caused by the 2012 Emilia earthquake sequence to industrial precast buildings, amongst other events. Building upon the concept of CCS, as documented in existing literature (e.g. Ref. [23]), a collection of inelastic spectra has been formulated. The primary aim is to obtain tools for expeditious numerical capacity/performance assessment of a precast reinforced concrete hinged frame, which could also be applicable during the initial stages of the design process. These spectra are generated through nonlinear IDA, making use of a set of 15 ground motion records obtained under far-field conditions on intermediate soil conditions. The analysis is conducted on ESDOF systems, the characteristics of which are derived from reference structures with shared geometric and mechanical attributes. The transition from the reference structure to the ESDOF system/model has been executed through a procedure rooted in DDBD, which approach also highlights the significant role of deformation in governing the seismic response of precast frames.

The pivotal findings of this research, whose methodological key aspects are briefly outlined above, can be enumerated as follows.

1. Two families of inelastic spectra are computed in this research: limit state spectra (LSS) and drift limit spectra (DLS). The former comprise collapse capacity (CC) spectra, damage limit (DL) spectra based on material strain, and serviceability limit (SL) spectra based on material strain. The latter include spectra calculated for drift limits of 0.5 % (DR05), 1.0 % (DR10), and 2.0 % (DR20). These spectra are instrumental in determining the response modification factor – see Equation (26) – concerning changes in the overall structure height $H_{n}$. The study delves into the influence of fundamental structural parameters, such as the number of storeys, columns' aspect ratio, stability index, and mass distribution along the height, each within a range of common variability.
2. Through comparative studies, it has been observed that the aspect ratio ($A_{r}$) exhibits minimal influence on the response modification factor in the limit state spectra (LSS). In contrast, $A_{r}$ plays a more pronounced role than the stability index (θ) in shaping the drift limit spectra (DLS). Conversely, the stability index (θ), in conjunction with the building height ($H_{n}$), emerges as the most critical parameter in determining the collapse capacity (CC) and damage limit (DL) capacities of the system. As θ increases, both CC and DL capacities tend to decrease, with their curves progressively converging. At θ equal to 0.3, the CC and DL values become nearly coincident.
3. The impact of mass distribution on spectral shapes has been determined to be relatively minor, with DLS that appears to be more sensitive to changes in roof mass configuration. A change in this parameter contributes only slight variations – of up to 10–20 % – to the overall trends, which are primarily governed by other structural parameters. Although variations with respect to the base case in which the mass at the roof coincides with that on the other floors tend to remain insensitive to the target limit state, slightly greater residuals can be observed as *R* increases (i.e. passing from SL to CC spectra).
4. Regression analyses of median spectra have been proposed, offering a valuable tool for the precise interpretation of the original data. More in detail, polynomial regression has generally fitted the inelastic spectra with good accuracy (i.e. 0.76 < *R*^*2*^ < 0.98) for most of the cases. Exception made for SL spectra regression, the *R*^*2*^ value is close to unity for CC spectra and lies around 0.75 in the other cases.

Additionally, it is noted that the plastic hinge hysteretic behaviour/model might exert an influence on the results, as inferred also by Adam and Jager [23]. While the use of the adopted Takeda cycle is generally deemed suitable for the modelling of plastic hinges in columns, this aspect warrants further in-depth study to comprehensively assess its impact. Finally, it is crucial to acknowledge that the structural stability index (θ) employed in this study is derived from Equation (10), which differs from the stability index equation found in building codes (i.e. Equation (1)). While it is feasible to compare the results with code provisions regarding stability indices, caution should be exercised due to these differences in the underlying equations.

In closing, it is worthy of mention that, in addition to issues related to changes in the uniaxial constitutive material model for the plastic hinge hysteretic behaviour, complete or on-spot checks/comparisons with counterpart three-dimensional model(s) of entire structure(s) could be non-negligible/trivial further developments, and the same applies with the derivation of other sets of inelastic performance-based spectra for other precast structures or precast technologies, although the repository provided in this piece of work remains quite vast. That stated, the latter research activity would simply call for further numerical analyses, undertaken in deterministic or probabilistic fashion to take into account and propagate uncertainties in geometry, material properties and loads, as the proposed framework could easily adapt to integrate all of the above items if/when fed with proper statistics and details/information. Nonetheless, the nature of numerical/analytical models and tools remains practice-oriented, and results could be of some little interest to practitioners for expeditious capacity assessment and/or pre-design checks, given also the current normative/codification context, as nonlinear time-history analyses are not required to be run for the design of such structures. This makes professional engineers – in Italy, at least – opt for linear elastic analysis as the favourite approach, which in turn makes the results proposed herein very informative. Time constraints and complete lack of design information/details – in the overwhelming majority of the cases – could very understandably complete the explanation for what concerns the case of field inspection endeavours in the aftermath of an earthquake.

## CRediT authorship contribution statement

**R. Nascimbene:** Writing – original draft, Supervision, Methodology, Conceptualization. **E. Brunesi:** Writing – review & editing, Supervision, Methodology, Conceptualization. **A. Sisti:** Investigation, Formal analysis, Data curation.

## Data and code availability statement

Data will be made available on request.

## Declaration of competing interest

The authors declare the following financial interests/personal relationships which may be considered as potential competing interests: The corresponding author, namely Emanuele Brunesi, serves as an Associate Editor of this journal. If there are other authors, they declare that they have no known competing financial interests or personal relationships that could have appeared to influence the work reported in this paper.

## Figures and Tables

**Table 4:** Regression coefficients for CC-LS3.

Coefficient	Mean value	${CI}_{95}$
$p_{00}$	17.74	(16.97, 18.51)
$p_{01}$	−188.9	(-207.8, −170)
$p_{02}$	766.8	(653.5, 880.2)
$p_{03}$	−1064	(-1275, −853)
$p_{11}$	1.804	(0.951, 2.658)
$p_{12}$	−3.961	(-6.4, −1.521)
$p_{20}$	−0.006086	(-0.008692, −0.00348)

**Table 5:** Regression indices for CC-LS3.

# Coeff. $p_{ii}$	$n_{df}$	RSS	$R^{2}$	STE
7	108	17.31	0.9797	0.4004

**Table 6:** Regression coefficients for DL-LS2.

Coefficient	Mean value	${CI}_{95}$
$p_{00}$	4.921	(4.62, 5.221)
$p_{01}$	−9.588	(-10.58, −8.595)
$p_{10}$	0.01329	(-0.008973, 0.03556)

**Table 7:** Regression indices for DL-LS2.

# Coeff. $p_{ii}$	$n_{df}$	RSS	$R^{2}$	STE
3	112	23.93	0.7664	0.4623

**Table 8:** Regression coefficients for SL-LS1.

Coefficient	Mean value	${CI}_{95}$
$p_{00}$	0.9961	(0.8449, 1.147)
$p_{10}$	0.01622	(-0.005705, 0.03815)
$p_{01}$	0.5209	(-0.3901, 1.432)
$p_{20}$	−0.0007148	(-0.001562, 0.0001329)
$p_{11}$	0.07567	(0.03166, 0.1197)
$p_{02}$	−2.529	(-4.673, −0.3852)

**Table 9:** Regression indices for SL-LS1.

# Coeff. $p_{ii}$	$n_{df}$	RSS	$R^{2}$	STE
6	109	0.673	0.4250	0.07858

**Table 10:** Regression coefficients for DR05.

Coefficient	Mean value	${CI}_{95}$
$p_{00}$	0.2234	(0.1966, 0.2502)
$p_{10}$	0.002707	(0.0007096, 0.004705)
$p_{01}$	−0.01665	(-0.01979, −0.0135)
$p_{11}$	−9.467e-005	(-0.0001743, −1.504e-005)
$p_{02}$	0.0003966	(0.0002966, 0.0004967)

**Table 11:** Regression indices for DR05.

# Coeff. $p_{ii}$	$n_{df}$	RSS	$R^{2}$	STE
5	455	0.691	0.7683	0.0123

**Table 12:** Regression coefficients for DR10.

Coefficient	Mean value	${CI}_{95}$
$p_{00}$	0.446	(0.3952, 0.4969)
$p_{10}$	0.005574	(0.003154, 0.007993)
$p_{01}$	−0.03329	(-0.03959, −0.027)
$p_{11}$	−0.000189	(-0.0003481, −2.987e-005)
$p_{02}$	0.0007933	(0.0005933, 0.0009932)

**Table 13:** Regression indices for DR10.

# Coeff. $p_{ii}$	$n_{df}$	RSS	$R^{2}$	STE
5	455	0.2762	0.7684	0.0246

**Table 14:** Regression coefficients for DR20.

Coefficient	Mean value	${CI}_{95}$
$p_{00}$	0.8927	(0.7911, 0.9943)
$p_{10}$	0.01115	(0.00631, 0.01599)
$p_{01}$	−0.06667	(-0.07925, −0.05408)
$p_{11}$	−0.000378	(-0.0006961, −5.992e-005)
$p_{02}$	0.001589	(0.001189, 0.001989)

**Table 15:** Regression indices for DR20.

# Coeff. $p_{ii}$	$n_{df}$	RSS	$R^{2}$	STE
5	455	1.1045	0.7686	0.0493

**Fig. 23:**
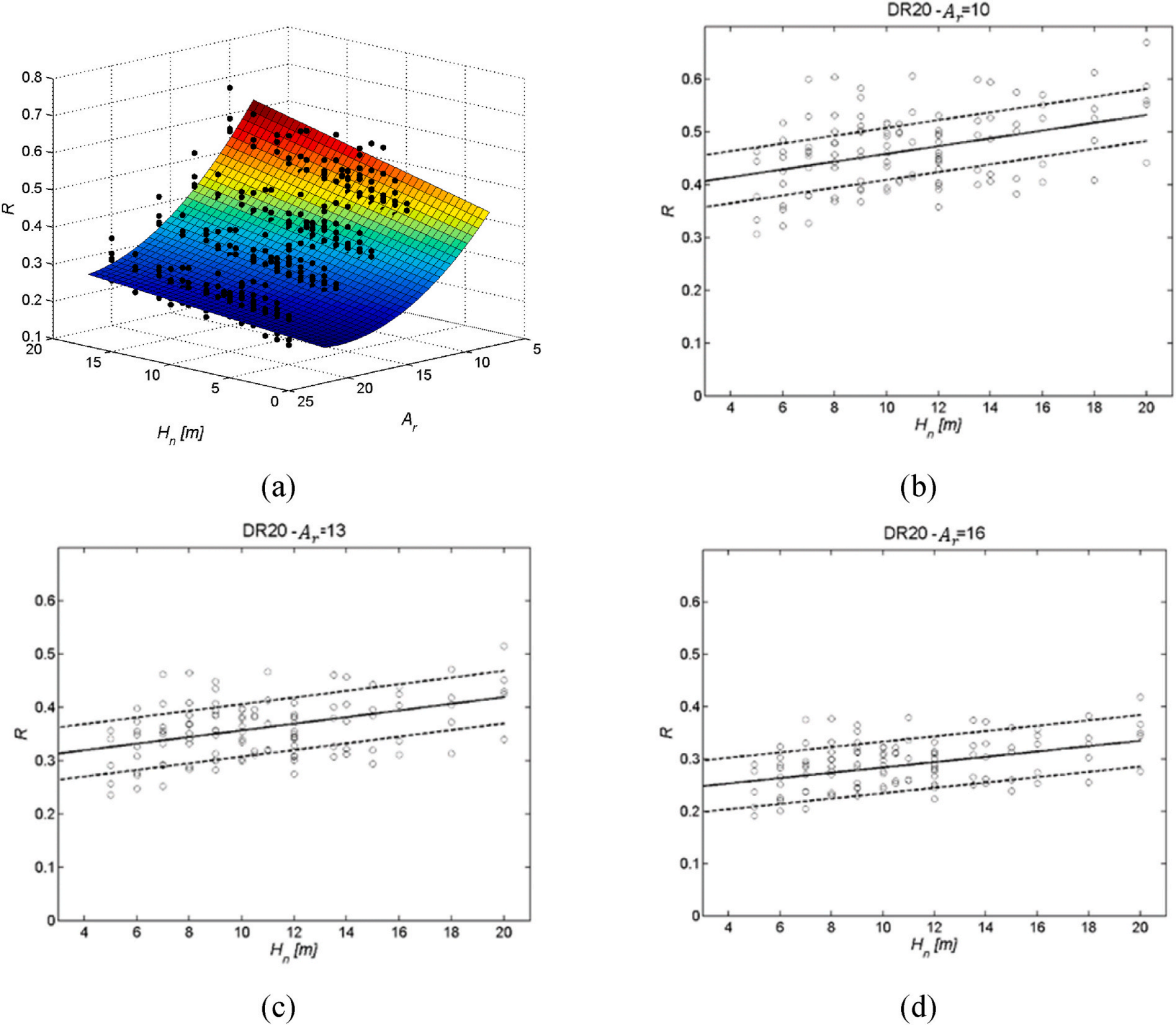
Regression for DR20: mean surface (top left) as a function of $H_{n}$, R, and $A_{r}$; single curves corresponding to $A_{r}=$ 10, 13, and 16. More specifically, (a) mean surface; (b) R versus $H_{n}$ for $A_{r}=$ 10; (c) R versus $H_{n}$ for $A_{r}=$ 13; (d) R versus $H_{n}$ for $A_{r}=$ 16.
